# Translational downregulation of Twist1 expression by antiproliferative gene, B-cell translocation gene 2, in the triple negative breast cancer cells

**DOI:** 10.1038/s41419-019-1640-z

**Published:** 2019-05-28

**Authors:** Preethi Devanand, Santhoshkumar Sundaramoorthy, Min Sook Ryu, Aravinth kumar Jayabalan, Takbum Ohn, In Kyoung Lim

**Affiliations:** 1 Department of Biochemistry and Molecular Biology, Ajou University School of Medicine, Suwon, 16499 Republic of Korea; 2 Division of Medical Sciences, Graduate School of Ajou University, Suwon, 16499 Republic of Korea; 3 BK21 Plus program, Department of Biomedical Sciences, Ajou University Graduate School of Medicine, Suwon, 16499 Republic of Korea; 4 Department of Cellular and Molecular Medicine, College of Medicine, Chosun University, Gwangju, 61452 Republic of Korea

**Keywords:** Breast cancer, Tumour-suppressor proteins

## Abstract

Twist1, a key transcription factor regulating epithelial–mesenchymal transition and cancer metastasis, is highly expressed in invasive cancers in contrast to the loss of BTG2^/TIS21^ expression. Based on our observation that forced expression of BTG2^/TIS21^ downregulated Twist1 protein expression without altering mRNA level, we investigated molecular mechanisms of the BTG2^/TIS21^-inhibited Twist1 translation in the triple negative breast cancer (TNBC) cells and in vivo BTG2^/TIS21^-knockout (KO) mice and human breast cancer tissues. (1) C-terminal domain of Twist1 and Box B of BTG2^/TIS21^ interacted with each other, which abrogated Twist1 activity. (2) BTG2^/TIS21^ inhibited translational initiation by depleting eIF4E availability via inhibiting 4EBP1 phosphorylation. (3) Expression of BTG2^/TIS21^ maintained p-eIF2α that downregulates initiation of protein translation, confirmed by eIF2α-AA mutant expression and BTG2^/TIS21^ knockdown in MEF cells. (4) cDNA microarray analysis revealed significantly higher expression of initiation factors-eIF2A, eIF3A, and eIF4G2-in the BTG2^/TIS21^-KO mouse than that in the wild type. (5) BTG2^/TIS21^-inhibited translation initiation lead to the collapse of polysome formation and the huge peak of 80s monomer in the BTG2^/TIS21^ expresser, but not in the control. (6) mRNAs and protein expressions of elongation factors were also downregulated by BTG2^/TIS21^ expression in TNBC cells, but much higher in both TIS21-KO mice and lymph node-positive human breast cancers. (7) BTG2^/TIS21^-mediated Twist1 loss was not due to the protein degradation by ubiquitination and autophagy activation. (8) Twist1 protein level was significantly higher in various organs of TIS21-KO mice compared with that in the control, indicating the in vivo role of *BTG2*^*/TIS21*^ gene in the regulation of Twist1 protein level. Altogether, the present study support our hypothesis that BTG2^/TIS21^ is a promising target to combat with metastatic cancers with high level of Twist1 without BTG2^/TIS21^ expression.

## Introduction

Epithelial–mesenchymal transition (EMT) is a process *trans*-differentiating epithelial cells to mesenchymal cells, which results in the increase of motility, migration, and invasion of cancer cells into extracellular matrix^1^. Metastasis of epithelial tumors takes place when the cancer cells lose cell–cell contact inhibition in contrast to obtaining migration capacity along with EMT process^2^. Twist1, a basic helix-loop-helix transcription factor, plays an important role in the embryonal development as a master regulator of morphogenesis^3^ and in EMT process during tumor invasion and metastasis^4^. Twist1 along with Bmi suppress the expression of E-cadherin^5^ in contrast to increasing N-cadherin expression by β1-integrin^6^, which promotes metastatic process. Twist1 expression induced by sunitinib accelerates vasculogenic mimicry and induction of cancer stem cell marker in triple negative breast cancer (TNBC) cells^7^.

In pathological context, initiation of cancer invasion requires actin polymerization by Dia1 and myosin recruitment to the fully matured focal adhesion site, which impose cells to get invasive motility rather than planar motility^8^. Among the several tumor suppressors, *BTG2*^*/TIS21*^ gene was primarily focused due to its targeting activity on actin nucleator, Dia1^9^. BTG2^10^ is a human ortholog of mouse TIS21^11^ and rat PC3^12^, which belongs to antiproliferative gene family. Expression of BTG2^/TIS21^ is frequently lost early in carcinogenesis^13–19^ by epigenetic change and miRNA regulation^20–22^. BTG2 helps CCR4-cNOT complex that degrades mRNAs and thereby inhibits target gene expression^23,24^. Overexpression of BTG2^/TIS21^ has been significantly associated with G2/M arrest by interacting with cyclin B1-Cdc2 complex^25^, and with cell death by inducing MnSOD expression via nuclear factor-κB activation^26,27^. BTG2^/TIS21^ high expression in breast cancer increases survival rate^28^, whereas loss of BTG2 expression induces breast tumor progression that responds to ErbB/HER inhibitor lapatinib^29^. Indeed, overexpression of BTG2^/TIS21^ inhibits invadopodia formation in the TNBC, MDA-MB-231, cells^30^ and reduces cancer invasion to muscle layer and lymph nodes of human bladder and breast cancers^9,20^. Constitutive expression of BTG2^/TIS21^ in the ductal carcinoma in situ (DCIS), but not in the infiltrating ductal carcinoma, strongly suggests that BTG2 might be an important barrier to block cancer progression from DCIS to infiltrating cancers^31^, and the notion is well supported by the report that p53 deficiency-enhanced metastatic potential of breast cancer is linked to BTG2^/TIS21^ loss in the primary and metastatic sites of TNBC-PDX model^32^.

In contrast to BTG2, Twist1 expression promotes invadopodia formation by inducing PDGFRα expression^33^ and exhibits poor prognosis in human breast cancer through Src co-expression^34^. We have screened in vitro protein interaction network of BTG2^/TIS21^ and observed Twist1 as one of the interacting partners of BTG2. Thus, we could hypothesize that BTG2^/TIS21^-inhibited cancer invasion might be through the downregulation of Twist1 activity in human breast cancers. To validate our hypothesis, adenoviral transduction of *BTG2*^*/TIS21*^ gene was employed in the TNBC cells and found the significant reduction of Twist1 translation, but not mRNA transcription, in the BTG2^/TIS21^ expresser along with the collapse of polysome formation on the Twist1 mRNAs. In the present study, we report potential mechanisms of the translational inhibition of *Twist1* gene by *BTG2*^*/TIS21*^ gene. To our best knowledge, this is the first report that BTG2^/TIS21^, a tumor suppressor, acts as a potential candidate to block EMT phenomenon by inhibiting Twist1 translation in TNBC cells.

## Results

### Downregulation of Twist1 protein expression by *BTG2*^*/TIS21*^ gene

**Fig. 1:**
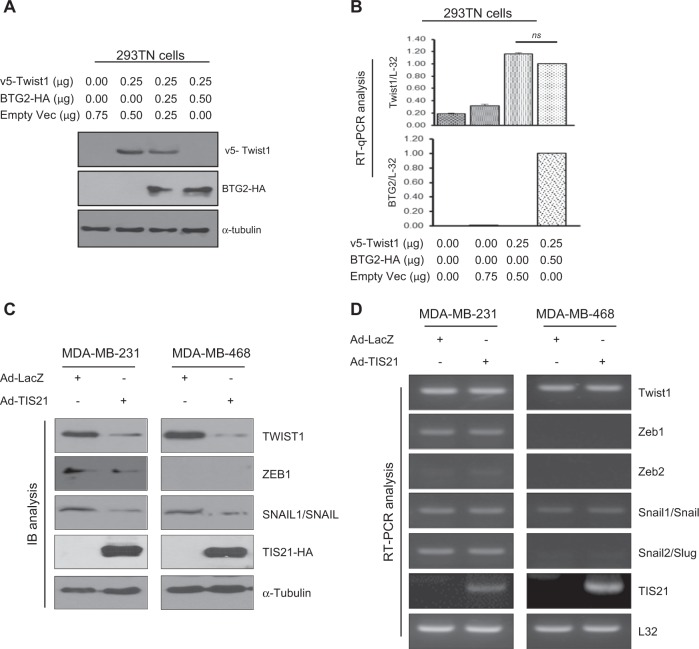
Expression of BTG2^/TIS21^ accompanies with loss of Twist1 protein, not mRNA, level. **a** When 293TN cells were transfected with v5-Twist1 for 48 h, its expression was significantly reduced in the dose-dependent manner of BTG2-HA. Anti-v5, anti-HA, and anti-α-tubulin antibodies were applied for immunoblot analysis. **b** To examine regulation of v5-Twist1 mRNA expression by BTG2-HA, real-time PCR analysis was performed at 48 h. L32 was used for loading control. Note the absence of significant difference in the Twist1 mRNA expression by BTG2-HA (**c**) MDA-MB-231 and MDA-MB-468 cells transduced with either Ad-LacZ (100 moi) or Ad-TIS21 (100 moi) for 48 h were subjected to immunoblot analysis and the significant loss of Twist1 expression along with ZEB and Snail was observed in the BTG2^/TIS21^ expresser. **d** The TNBC cells were subjected to RT-PCR analysis and mRNAs levels were measured. Note absence of significant changes in the levels of Twist1, Zeb1, and Snail between the BTG2^/TIS21^ and the LacZ expressers. Immunoblots (**a**, **c**, **d**) are representative of three independent experiments. Data are expressed as mean ± SD from the more than two independent experiments

To explore regulation of Twist1 expression by *BTG2*^*/TIS21*^ gene, transfection analysis was performed in 293TN cells for 48 h with *v5-Twist1* and *BTG2-HA* gene, and the cells were analyzed by immunoblotting. Twist1 protein expression was significantly downregulated by BTG2^/TIS21^ expression (Fig. 1a) without any significant change in the Twist1 mRNA level (Fig. 1b). Initially, we screened various breast cancer cells with and without invasiveness to identify if BTG2 and Twist1 expressions are mutually exclusive. As shown in Supplementary Fig. S1A, we found that MCF-7 and ZR-75-1 cells expressed endogenous BTG2 without TWIST1 expression. The finding was opposite in the TNBC cells with high TWIST1, but no BTG2, expression. To explore whether the amount of exogenous BTG2^/TIS21^ expressed in the TNBC cells by adenoviral transduction is relevant to physiological level or not, immunoblot analysis was performed and confirmed that the exogenous BTG2^/TIS21^ level was about physiologically relevant (Supplementary Fig. S1B). To further examine whether endogenous BTG2 protein can also regulate Twist1 expression or not, MCF-7 cells were employed and the endogenous BTG2 expression was knocked down by siBTG2 transfection. As expected, the transfection significantly induced TWIST1 protein level, but not Twist1 mRNA, in the MCF-7 cells (Supplementary Fig. S1C). In addition to Twist1, Zeb1 and Snail protein expressions were also regulated by BTG2^/TIS21^ expression (Fig. 1c), whereas their mRNA expressions were not altered (Fig. 1d). BTG2^/TIS21^-regulated TWIST1 protein level was dependent on the concentration of BTG2^/TIS21^ gene (Supplementary Fig. S2A). As BTG2^/TIS21^ downregulates interleukin (IL)-6 mRNA expression^35^, IL-6 transcription was monitored as a control experiment to prove our observation. As expected, it was significantly reduced in both TNBC cells with BTG2^/TIS21^ expression (Supplementary Fig. S2B). To validate the phenomenon in the MDA-MB-231 cells depleted with serum for 22 h, fetal bovine serum (FBS; 0–10%) was supplemented for 6 h before immunoblot analysis. As expected, Twist1 biosynthesis was able to be induced in the LacZ expresser by serum stimulation in the concentration- and the time-dependent manners, whereas it was not in the BTG2^/TIS21^ expresser (Supplementary Fig. S2C and S2D). To screen whether BTG2^/TIS21^ downregulated poly(A) tail length of Twist1 mRNA or not, poly(A) tail assay (PAT) was performed. Twist1 mRNA and poly(A) tail length unchanged by transduction of MDA-MB-231 cells with Ad-BTG2^/TIS21^ (Supplementary Fig. S3). The data support that the BTG2^/TIS21^-mediated downregulation of EMT factors might be at the protein, but not mRNA, levels.

### Inhibition of Twist1 activity by *BTG2*^*/TIS21*^ gene in the TNBC cells

**Fig. 2:**
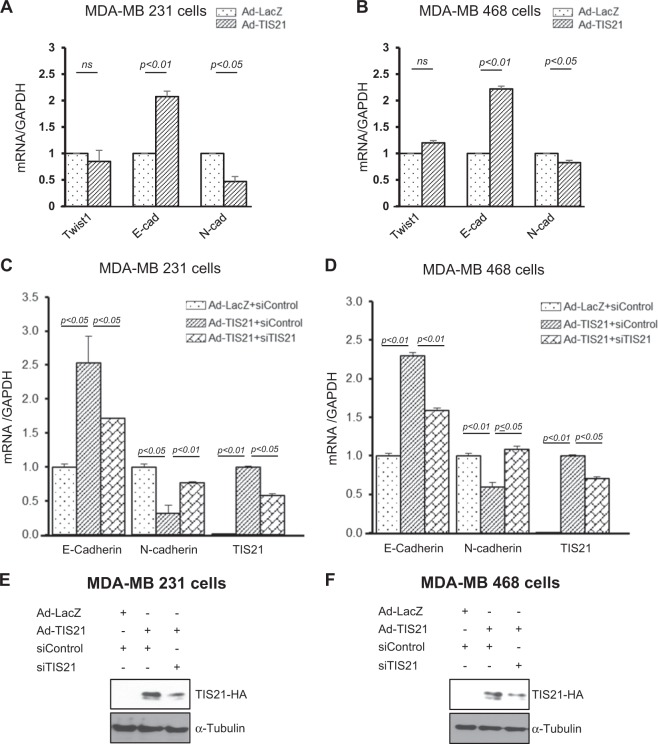
Knockdown of BTG2^/TIS21^ expression recovers Twist1 activity. **a** Regulation of Twist1 expression by *BTG2*^*/TIS21*^ gene was re-examined via measuring its target gene expression by real-time PCR after forced expression of *BTG2*^*/TIS21*^ gene in MDA-MB-231 cells. Note the reciprocal regulation of E-cadherin and N-cadherin expressions without significant change of Twist1 mRNA expression by *BTG2*^*/TIS21*^ gene. GAPDH served as a control for amplification. **b** The same experiment was performed in MDA-MB-468 cells and similar regulation was observed in the BTG2^/TIS21^ expresser. **c** To confirm the activity of *BTG2*^*/TIS21*^ gene in the regulation of Twist1 target genes, MDA-MB-231 cells were transfected with siControl or siTIS21 (100 nM) for 24 h, and then transduced with either Ad-LacZ or Ad-TIS21 (100 moi). Total cellular RNAs were isolated 24 h later and subjected to real-time PCR analysis, and the expressions of E-cadherin, N-cadherin, and BTG2^/TIS21^ were measured along with GAPDH as an internal control. *p* < 0.05 was considered as statistically significant. Note the significant changes of E-cadherin and N-cadherin expressions by the level of *BTG2*^*TIS21*^ gene expression. **d** The same experiment was performed in MDA-MB-468 cells and the similar regulation of Twist1 target gene expression by BTG2^TIS21^ was also found in the cells. Immunoblot analyses reveal the knockdown efficiency of siTIS21 in the MDA-MB-231 (**e**) and MDA-MB-468 (**f**) cells. Anti-HA and anti-α-tubulin antibodies were applied for the experiment (*n* = 2). All data are expressed as mean ± SD after two independent experiments

To further examine whether the BTG2^/TIS21^-mediated Twist1 protein loss downregulates expression of Twist1 targets genes, E-cadherin and N-cadherin levels were measured by reverse transcriptase-quantitative PCR (RT-qPCR). Despite no significant change in the Twist1 mRNA expression, E-cadherin and N-cadherin expressions were significantly altered in the BTG2^/TIS21^ expresser compared with those in the LacZ control (Fig. 2a, b). To further evaluate specificity of the regulation, expression of BTG2^/TIS21^ was knocked down by siTIS21 transfection and the recovery of the target gene expressions was observed in both TNBC cells (Fig. 2c, d). Efficiency of short interfering RNA (siRNA) knockdown was confirmed by immunoblot analysis with anti-hemagglutinin (HA) and anti-α-Tubulin antibodies (Fig. 2e, f).

### In vitro interaction of BTG2-box B with C-terminal region of Twist1

**Fig. 3:**
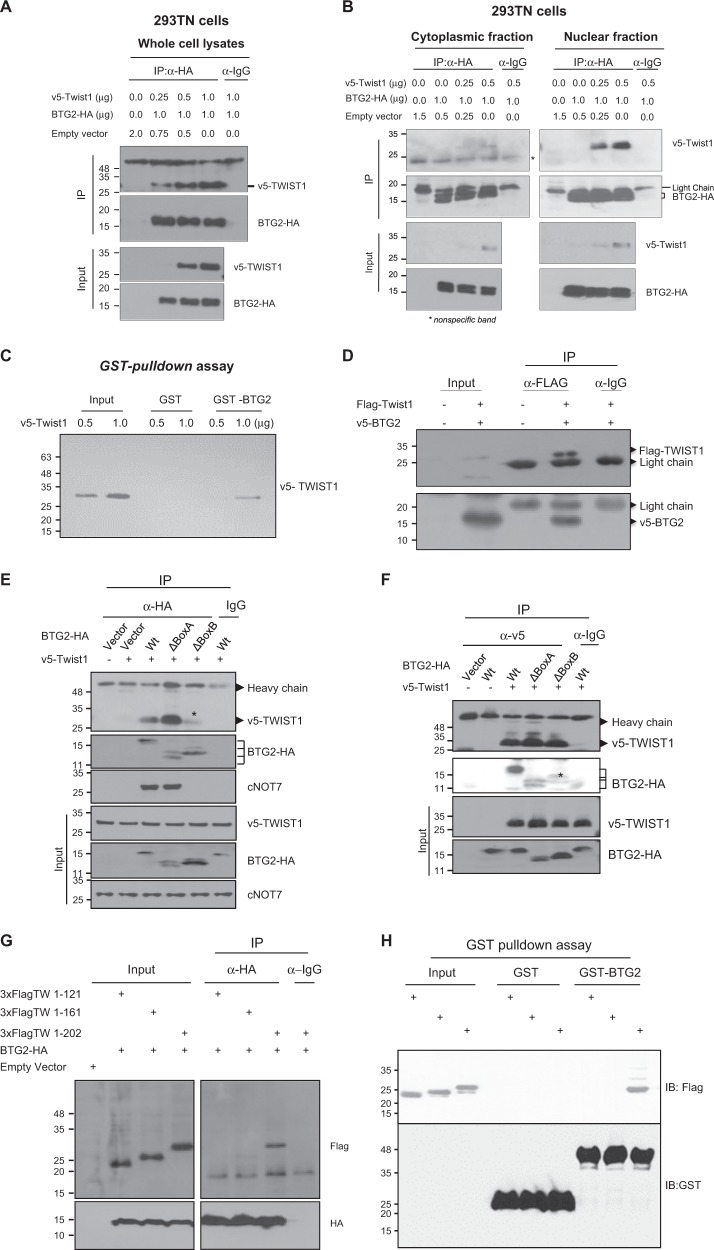
C-terminal region of TWIST1 interacts with BTG2 box B domain. **a** In vitro immunoprecipitation (IP) analysis was performed in 293TN cells with anti-HA antibody. Pulldown of BTG2-HA using 1.0 µg of anti-HA antibody showed its interaction with v5-Twist1, depending on its concentration. Normal IgG was employed as a negative control of IP assay. Immunoblotting is the representative of three independent experiments. **b** Fractionation of the cytoplasm and nucleus of 293TN cells transfected with v5-Twist1 and BTG2-HA was performed, and the interaction of Twist1 and BTG2 was further examined by IP with 1.0 µg of anti-HA antibody. The data suggest that interaction was mainly observed in the nuclear fraction than in the cytoplasm. Star indicates nonspecific band. **c** In vitro GST-pulldown assay. GST and GST-BTG2 beads were incubated with 293TN cell lysates transfected with v5-Twist1 at 4 °C overnight. The beads were pulled down and subjected to immunoblot analysis with anti-v5 antibody. Note the direct interaction of BTG2 with v5-Twist1. **d** IP analysis was performed in 293TN cells after transfection with Flag-Twist1 (1.0 μg) and v5-BTG2 (1.0 μg) genes. In vitro interaction of Flag-Twist1 and v5-BTG2 was able to be shown by IP with 1.0 µg of anti-Flag antibody. Immunoblotting is the representative of three independent experiments. **e** For mapping BTG2 domain bound to Twist1, 293TN cells were transfected with either BTG2-HA wild type or deletion mutants (ΔBox-A, ΔBox B) along with *v5-Twist1* gene. IP analysis performed with anti-HA antibody found that overexpression of ΔBox-B mutant significantly reduced BTG2-Twist1 interaction, indicating that box B in BTG2-HA interacted with v5-Twist1 protein. *The reduced interaction between BTG2-HA and v5-Twist1. BTG2 with ΔBox-B also abrogated interaction with endogenous cNOT7. **f** IP analysis with anti-v5 antibody. Procedure was the same as described in the **e**. Note the star revealing significant loss of the interaction. **g** To investigate the domain in Twist1 interacting with BTG2-HA, 293TN cells were transfected with either Twist1 wild type (1–202) and its deletion constructs (1–121, 1–161) along with BTG2-HA. IP performed with anti-HA antibody showed that the wild-type Twist1, but not the deletion mutants, could interact with BTG2-HA. **h** In vitro interaction analysis by GST-pulldown assay. GST and GST-BTG2 beads were incubated with 293TN cell lysates transfected with either wild type or the deletion constructs of *Twist1* gene at 4 °C overnight. When the beads were subjected to immunoblot analysis with anti-Flag antibody, the interaction of BTG2 with wild-type Twist1, but not deletion mutants, was observed. All data clearly indicate the in vitro interactions of C-terminal region of Twist1 with BTG2 box B domain. Blots (**b**, **c**, and **e**–**g**) are representative of two independent experiments

BTG2 exerts its activity by interacting with biologically significant proteins such as Hoxb9^36^, rPICK1^37^, cNOT7/8^23,24^, PRMT1^38^, and Pin-1^39^. By using 19K-protein chip analysis, various proteins interacting with BTG2^/TIS21^ protein could be isolated by Ho Chul Kang at Ajou University, BTG2-HA interacted with v5-Twist1 dose dependently (Fig. 3a), and the interaction occurred mainly in the nucleus rather than the cytoplasmic fraction (Fig. 3b). It has been known that cNOT7 binds to BTG2^23,24^; thus, competition assay was performed with constant amounts of BTG2-HA and v5-Twist1 along with the increasing amount of Flag-cNOT7 construct. Immunoprecipitation (IP) with anti-BTG2-HA antibody revealed strong interaction of BTG2-HA with v5-TWIST1, independent of the cNOT7 in the whole-cell lysates of 293TN cells (Supplementary Fig. S4A). When fractionation was applied, cNOT7 was expressed in the cytoplasm, but TWIST1 was in the nuclear fractions (Supplementary Fig. S4B). GST-pulldown analysis also exhibited in vitro interaction of v5-Twist1 with GST-BTG2 (Fig. 3c). To exclude the effect of protein tag on their interaction, the reciprocal IP was performed and observed the interaction of Flag-Twist1 with v5-BTG2 in 293TN cells (Fig. 3d). As intracellular location of Twist1 expression has been reported as variable in different cancer cells^40,41^, HeLa cells were employed and the cytoplasmic expression of Twist1 was found (Supplementary Fig. S4C); the phenomenon was further confirmed by confocal analysis (Supplementary Fig. S4D). When competition assay was performed in HeLa cells, the expression of Flag-cNOT7 significantly reduced v5-Twist1 binding to BTG2-HA concentration dependently (Supplementary Fig. S4E). To identify BTG2 domain interacting with Twist1, reciprocal IP analyses were performed. The Box B-deleted BTG2 mutant significantly abrogated BTG2 binding with v5-Twist1 and with cNOT7 (Fig. 3e, f), indicating the interaction of BTG2-Box B with TWIST1 and cNOT7. For mapping the domain of Twist1 protein interacting with BTG2, 3xFlag-tagged Twist1 mutants (1–121 and 1–161) and its wild-type (Wt) construct were co-transfected with BTG2-HA and then evaluated by IP analysis. Fig. 3g showed that only the full-length Twist1, but not the deletion mutants, could strongly bind to BTG2-HA. The data were confirmed by in vitro GST-pulldown assay with the clear interaction of BTG2 only with the full-length Twist1 (Fig. 3h). The data were further supported by the prediction of intrinsically unstructured protein analysis that exhibits only the C-terminal region (121–202 amino acid) of Twist1 forms ordered structure (Supplementary Fig. S4F), whereas the whole regions of BTG2 and TIS21 proteins form the globular structure (Supplementary Fig. S4G and S4H). To further investigate whether endogenous BTG2 also interacts with TWIST1, v5-Twist1 was transfected into MCF-7 cells and subjected to IP with anti-BTG2 antibody. Endogenous BTG2 protein clearly interacted with v5-TWIST1 and with cNOT7 as shown in Supplementary Fig. S5A. To analyze localization of the proteins in MCF-7 cells, fractionation was performed and we observed cNOT7 only in the cytoplasm; however, BTG2 and v5-TWIST1 proteins were more in the nuclear fraction than in the cytoplasm (Supplementary Fig. S5B).

### Proteasome- and lysosome-independent loss of TWIST1 protein by *BTG2*^*/TIS21*^ gene

**Fig. 4:**
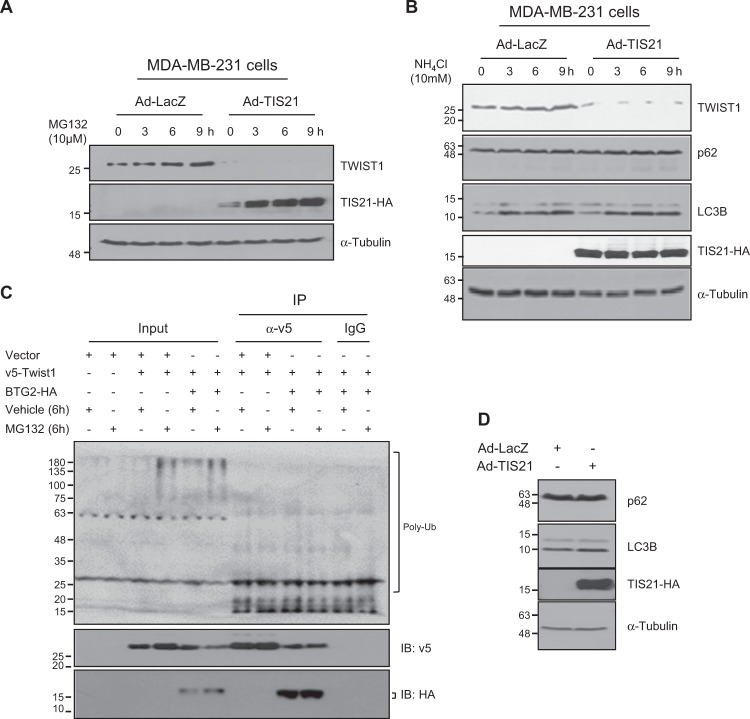
BTG2^/TIS21^-induced loss of TWIST1 expression is not due to protein degradation. **a** MG132 treatment failed to protect the BTG2^/TIS21^-induced Twist1 loss. MDA-MB-231 (3 × 10^5^/60 mm dish) cells were transduced with either Ad-LacZ (100 moi) or Ad-TIS21(100 moi) virus for 48 h and then treated with MG132 (10 µM) for various time points to block proteasome activity. Twist1 protein was accumulated by the treatment in the LacZ expresser, whereas it was failed in the BTG2^/TIS21^ expresser as opposed to accumulation of BTG2^/TIS21^ expression that served as a positive control. α-Tubulin served as a loading control. **b** BTG2^/TIS21^-induced Twist1 loss cannot be protected by NH_4_Cl treatment. MDA-MB-231 (3 × 10^5^ cells) cells transduced with either Ad-LacZ or Ad-TIS21 were treated with 10 mM of NH_4_Cl to block lysosome activity before immunoblot analysis at the time points. Twist1 protein was accumulated in the LacZ expresser by the treatment; however, it was lost in the BTG2^/TIS21^ expresser. NH_4_Cl treatment also failed to alter the expression of p62. NH_4_Cl increased LC3B, the autophagosome-associated lipid form of LC3, which accumulates if their lysosomal degradation is inhibited, which served as a positive control. α-Tubulin served as a loading control. **c** Ubiquitination analysis. To investigate whether BTG2^/TIS21^-induced TWIST1 loss is regulated by ubiquitination or not, 293TN cells were transfected with either v5-Twist1 (1.0 μg) and/or BTG2-HA (1.0 μg) before IP analysis with anti-v5 antibody and immunoblot assay with anti-ubiquitin antibody. v5-Twist1 expression was slightly increased in the vector expresser by MG132 treatment (compare lane 4 vs. lane 3 in the v5-panel); however, it was rather decreased in the BTG2^/TIS21^ expresser (compare lane 6 vs. lane 5 in the v5 panel). The interaction between v5-Twist1 and BTG2^/TIS21^-HA was almost similar and there was no difference in the ubiquitination after MG132 treatment. **d** No alteration of autophagy signal in the BTG2^/TIS21^ expresser vs. LacZ control. MDA-MB-231 cells transduced with either Ad-LacZ or Ad-TIS21 were subjected to immunoblot analysis, to examine p62 and LC3B conversion. α-Tubulin as loading control. Blots are a representative of two independent experiment

MDA-MB-231 cells with BTG2^/TIS21^ or LacZ overexpression were incubated with either MG132 (proteasome inhibitor) or ammonium chloride (lysosome inhibitor) for 9 h and the change of TWIST1 protein was measured at every 3 h along with the TIS21-HA and LC3B accumulation as the positive controls; the inhibitions of proteasome and lysosome activity failed to block the BTG2^/TIS21^-mediated Twist1 loss (Fig. 4a, b). To examine whether Twist1 ubiquitination is enhanced by BTG2^/TIS21^, MDA-MB-231 cells transfected with 3xFlag-Twist1 with or without BTG2-HA were examined by IP and immunoblot analysis; ubiquitination of Twist1 protein was not enhanced in the BTG2^/TIS21^ expresser (Supplementary Fig. S6A), and that was similar in the 293TN cells transfected with v5-Twist1 with or without BTG2-HA under MG132 treatment (Fig. 4c). We have recently reported that ABI-2 is targeted to proteasome-mediated degradation by BTG2^42^. Hence, ABI-2 was employed as a positive control of proteasome activation and confirmed that BTG2^/TIS21^ expression slightly increased ubiquitination of ABI-2 protein along with its interaction (Supplementary Fig. S6B). When the onset of autophagy program upon Ad-BTG2^/TIS21^ transduction was examined, the expression of p62 and LC3B conversion were not significantly changed (Fig. 4d).

### BTG2^/TIS21^-mediated Twist1 loss via downregulating translational initiation of protein biosynthesis

**Fig. 5:**
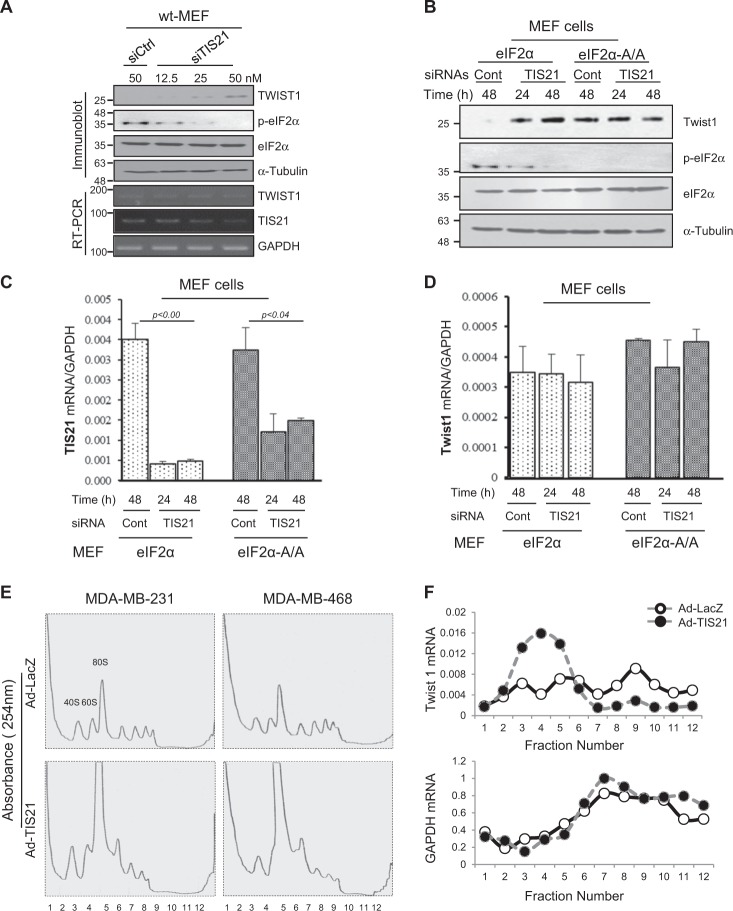
BTG2^/TIS21^-induced Twist1 loss is associated with the failure of polysome formation. **a** Twist1 expression was significantly induced by knockdown of endogenous BTG2^/TIS21^ expression. To confirm the effect of BTG2^/TIS21^ on the Twist1 protein loss, TIS21^/BTG2^ expression in the wild-type MEF was removed by transfection with siTIS21 (12.5~50 nM) for 2 days and then analyzed by immunoblotting with anti-Twist1 antibody. The induction of Twist1 expression appeared in the siTIS21-dependent manner, compared with that in the control. In the same cells, p-eIF2α level maintained in the siControl-transfected cells was significantly reduced by knockdown of BTG2^/TIS21^ expression. Total eIF2α levels remained unaltered. α-Tubulin served as a loading control. RT-PCR analysis showed that Twist1 mRNA level was unchanged. TIS21 knockdown was analyzed by RT-PCR and GAPDH served as a control. **b** Wild-type MEF cells overexpressed with either eIF2α or eIF2α-A/A (S^51^A/S^51^A) mutant were transfected with siTIS21-RNAs (50 nM) and siControl-RNAs (50 nM), and the cells were subjected to immunoblot assay in 24 and 48 h of the transfection. Knockdown of BTG2^/TIS21^ expression by siTIS21 significantly induced Twist1 expression compared with that in the siControl (lanes 2 and 3 vs. lane 1); however, Twist1 expression was consistently high in the MEF cells with eIF2α-A/A mutant expresser (lanes 5 and 6 vs. lane 4). The phosphorylation of eIF2α completely disappeared by the knockdown of BTG2^/TIS21^ expression for 48 h, suggesting that BTG2^/TIS21^ expression might maintain the p-eIF2α level that inhibits translation of Twist1. **c**, **d** Real-time qPCR analyses revealing mRNA expressions of BTG2^/TIS21^ (**c**) and Twist1 (**d**) in the MEF cells transfected with wild-type and mutant eIF2α. Transfection of MEF with siTIS21 markedly reduced BTG2^/TIS21^ expression; however, Twist1 transcription was not significantly changed by BTG2^/TIS21^ knockdown. Relative expressions were presented based on that of GAPDH. **e** Polysome profiling assay; to assess a general translational control by *BTG2*^*/TIS21*^ gene, TNBC cells transduced with either Ad-LacZ or Ad-TIS21 for 48 h were subjected to polysome profiling analysis. Polysome formation in the BTG2^/TIS21^ expresser was collapsed after a huge peak of the 80S monomer, whereas the profile was well maintained in the LacZ control. Numbers in *X* axis (1–12) indicate fractions collected up to 500 μL per each tube. **f** Real-time PCR analysis; to analyze abundance of the Twist1 mRNAs in each fraction, real-time PCR analysis was performed. The amount of Twist1 mRNA was higher in the 2–5 fractions of the BTG2^/TIS21^ expresser; however, it was much less in the 6–12 fractions compared with those in the LacZ expresser. In contrast, levels of GAPDH mRNA were similar between the LacZ and BTG2^/TIS21^ expressers in each fraction. Blots are representative of three independent experiments. All data are expressed as mean ± SD after two independent experiments

To further investigate whether the BTG2^/TIS21^-mediated Twist1 loss is ascribed to the translational defect, endogenous BTG2^/TIS21^ expression was knocked down by siRNAs to TIS21 (siTIS21, 0–50 nM) in the Wt MEF cells, and found the induction of TWIST1 expression by siTIS21 transfection along with the loss of p-eIF2α without eIF2α protein change (Fig. 5a). When the endogenous TIS21 expression was knocked down, TWIST1 protein level was clearly increased without change in its mRNA levels (Fig. 5a). To explore whether BTG2^/TIS21^ regulates Twist1 translation at initiation phase, MEF cells were overexpressed with either Wt eIF2α or mutant eIF2α (eIF2α-Α/Α) for 48 h. Knockdown of TIS21 expression significantly induced TWIST1 protein along with the loss of p-eIF2α (Fig. 5b, lanes 1–3). In contrast to wt-MEF, mutant MEF with eIF2α-A/A cannot be phosphorylated as shown in Fig. 5b. The data suggest that BTG2^/TIS21^ might inhibit TWIST1 biosynthesis through maintaining eIF2α phosphorylation, whereas Twist1 mRNA expression was not changed by knockdown of BTG2^/TIS21^ expression in the MEF cells (Fig. 5c, d), implying that BTG2^/TIS21^ downregulates Twist1 translation at the initiation phase by inhibiting eIF2α activity via inducing its phosphorylation in normal cells. The notion can be supported by the reports that homozygous mutant eIF2α-Α/Α fails to attenuate protein synthesis under stress condition^43^ and phosphorylation of eIF2α on S^51^ residue cannot form ternary complex to pre-initiate protein translation^44^. In TNBC cells, there was no physical interaction between BTG2^/TIS21^ and eIF2α molecules (Supplementary Fig. S7A and S7B). Moreover, transduction of TNBC cells with Ad-BTG2^/TIS21^ significantly inhibited phosphorylation of 4EBP1 at S^65^ and at T^37/46^, but not T^70^, residues (Supplementary Fig. S7C and S7D), which is the essential step to activate 4EBP1^45,46^. Phosphorylation of T^37^ and T^46^ has been reported as a priming event that permits phosphorylation of 4EBP1 at S^65^ residue that promotes dissociation of eIF4E from p-4EBP1 and permits formation of a functional eIF4F complex^45^. In addition, our cDNA microarray data (NCBI Gene Expression Omnibus as GSE 105772) exhibited significantly elevated expression of the initiation factors in the TIS21-KO mice than those in the wt mice (Supplementary Fig. S8). To validate the notion in detail, we performed polysome profiling assay in the TNBC cells and observed that BTG2^/TIS21^ expresser revealed collapse of the 80s polysome formation (fraction 6–10) along with the huge single peak of 80S monosome (fraction 5, Fig. 5e). To further confirm the phenomenon, abundance of Twist1 mRNA on the ribosomes was determined by real-time PCR analysis. Twist1 mRNA level was much lower in the BTG2^/TIS21^ expresser than that in the LacZ control (upper panel, Fig. 5f), whereas GAPDH mRNA level was not different in the two expressers (lower panel, Fig. 5f). As mTORc1 activity can be inhibited by BTG2^/TIS21^ expression^31^ and Twist1 mRNA contains 5′TOP motif (Supplementary Fig. S9A), we treated the MDA-MB-231 cells with Rapamycin, Torin, and PP242 for 6–12 h and observed that TWIST1 protein was significantly reduced by mTORc inhibition (Supplementary Fig. S9B). Collectively, the expression of BTG2^/TIS21^ downregulated initiation of Twist1 translation in the TNBC cells.

### Attenuated expression of eukaryotic elongation factors by *BTG2*^*/TIS21*^ gene

**Fig. 6:**
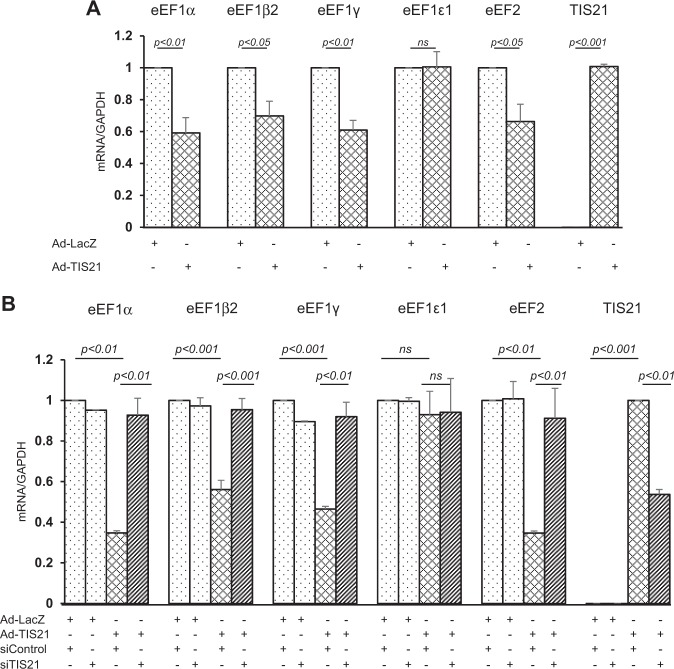
Downregulation of elongation factor expression by *BTG2*^*/TIS21*^ gene in MDA-MB-231 cells. **a** When the cells transduced with either Ad-LacZ or Ad-TIS21 were analyzed by real-time PCR, mRNA expressions of eEF1α, eEF1β2, eEF1γ, and eEF2 were significantly reduced in the BTG2^/TIS21^ expresser than those in the LacZ control. GAPDH served as an internal control of transcription. All data are expressed as mean ± SD after three independent experiments. **b** To validate the activity of *BTG2*^*/TIS21*^ gene regulating the transcription of elongation factors, knockdown of the exogenously expressed *TIS21* gene was performed by transfection with either siControl or siTIS21 (100 nM) for 24 h after adenovirus transduction for 24 h. Real-time PCR analysis showed significant recovery of the BTG2^/TIS21^-induced transcriptional repression by TIS21 knockdown in the cells. GAPDH served as an internal control. *p* < 0.05 was considered as statistically significant. All data are expressed as mean ± SD after two independent experiments

**Fig. 7:**
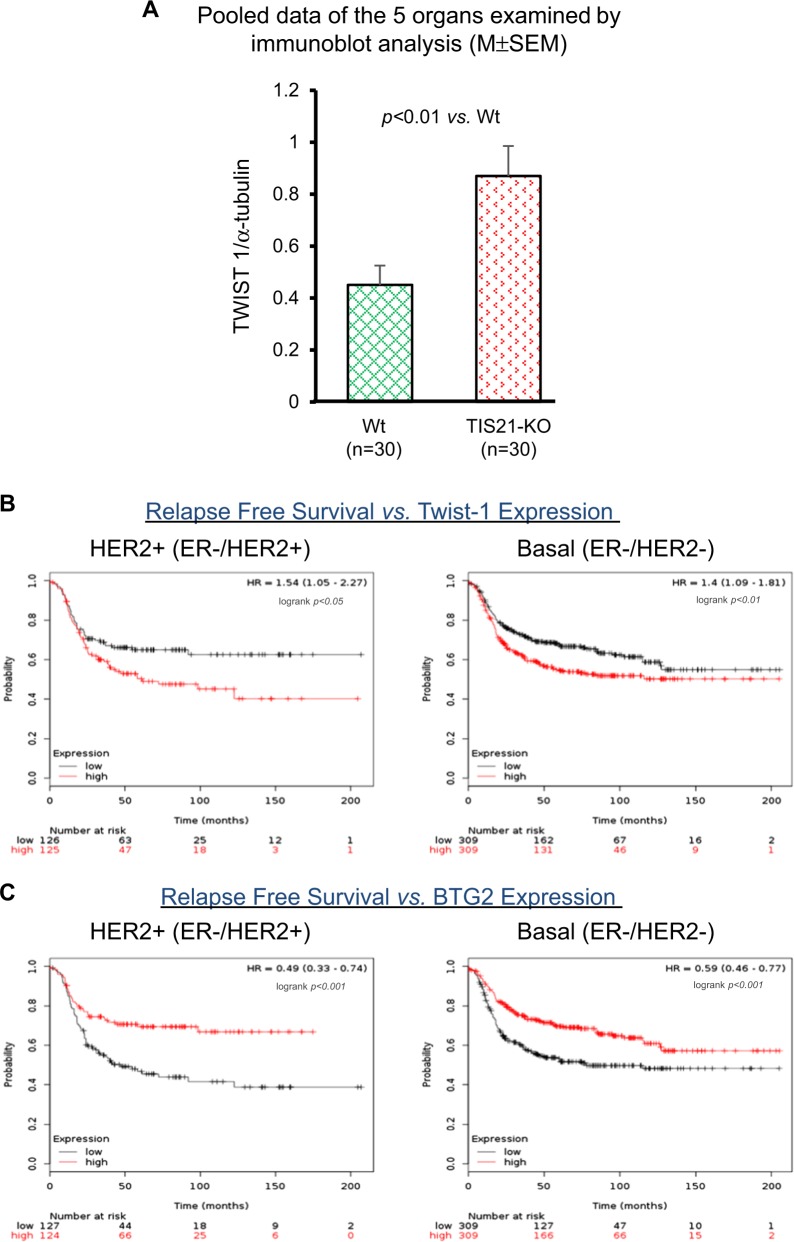
In vivo activity of *BTG2*^*/TIS21*^ gene in the TIS21-knockout mice and human breast cancers. **a** To analyze in vivo regulation of Twist1 expression by *BTG2*^*/TIS21*^ gene, five organs were extracted from each TIS21-KO (six heads) and the WT (six heads) female mice, and then subjected to immunoblot analyses with 40 µg/lane in each sample. When the ratios of Twist1/α-tubulin expression were examined by Image J software, the level was significantly higher in the TIS21-KO mice than that in the WT (*n* = 30 organs in each group). **b** Relapse-free survival vs. Twist1 expression was analyzed from open data in the HER2+ and Basal-type breast cancers. Higher expression of Twist1 significantly decreased the relapse free survival of HER2+ (*p* < 0.05) and Basal-type (*p* < 0.01) breast cancers. **c** Relapse-free survival vs. BTG2 expression was analyzed from open data in the HER2+ and Basal-type breast cancers. Higher expression of BTG2 significantly increased the relapse free survival of the HER2+ (*p* < 0.001) and Basal-type (*p* < 0.001) breast cancer patients

As polysome formation of Twist1 mRNA was significantly reduced by BTG2^/TIS21^, expression of elongation factors (eEFs) was measured in the TNBC cells by real-time PCR analysis and observed the significant reduction of eEF1α, eEF1β2, eEF1γ, and eEF2, except eEF1ε1 mRNAs in the BTG2^/TIS21^ expresser (Fig. 6a). However, knockdown of BTG2^/TIS21^ expression by siTIS21 transfection significantly recovered eEFs mRNA levels (Fig. 6b) and transduction with Ad-BTG2^/TIS21^ abolished eEF2 protein expression (Supplementary Fig. S7D). In addition, the expressions of eEF1γ and eEF2 were higher in the liver of the TIS21-KO mice, and eEF1α, eEF1β2, and eEF1γ were higher in the lung and spleen of the TIS21-KO mice than those in the wt mice (Supplementary Fig. S10C–S10E) without any significant alterations in the body and organ weights of the mice (Supplementary Fig. S10A, S10B). In vivo Twist1 mRNA expressions between the TIS21-KO and the wt mice were not statistically different (Supplementary Fig. S10F and S10G); however, in vivo Twist1 protein level in the lung and spleen was higher in the TIS21-KO mice compared with that in the Wt mice (Supplementary Fig. S11). When the expression ratio of Twist1/α-tubulin was pooled from the 30 organs dissected from six mice, Twist1 protein level was significantly higher in the TIS21-KO mice than that in the Wt evaluated by Mann–Whitney *U*-test (Fig. 7a). Above data strongly supported in vivo effect of *BTG2*^*/TIS21*^ gene on the translation of Twist1 mRNA. To further estimate the role of BTG2^/TIS21^ playing in human breast cancer tissues, lymph node-negative and -positive tumor samples and the matched surrounding normal tissues were analyzed and found the active expressions of TWIST1, SNAIL, and eEF2 in the node-positive tumors compared with the node-negative tissues. BTG2 expression was detectable only in the normal tissues (Supplementary Fig. S12). The data can be supported by the report that high expression of eEF2, regulating the rate of peptide chain elongation during protein translation, was significantly associated with node positivity and tumor size in breast cancers^47^. In addition, relapse-free survival rates of breast cancers (both HER2^+^ and HER2^−^) were negatively correlated with the level of Twist1, but positively correlated with BTG2 expression by open data analysis (Fig. 7b, c).

## Discussion

**Fig. 8:**
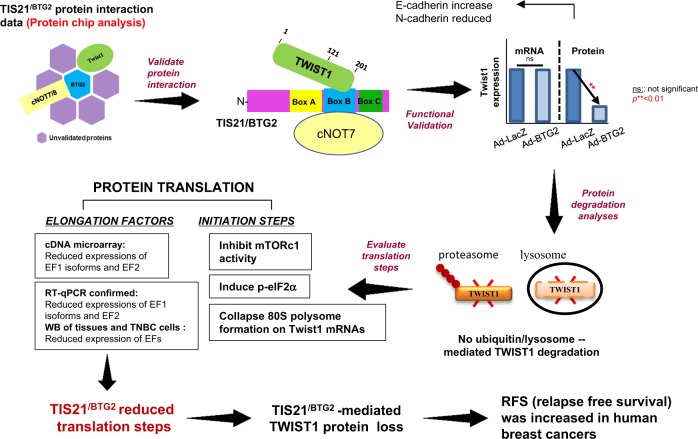
Summary depicting the effects of *BTG2*^*/TIS21*^ gene on the downregulation of Twist1 translation. Reciprocal immunoprecipitation analysis confirmed the interaction of C-terminal of Twist1 to BTG2^/TIS21^ box B domain, which had been screened by protein chip analysis. In the present assay, cNOT7 binding to BTG2^/TIS21^ was employed as the interaction control. Adenoviral transfer of *BTG2*^*/TIS21*^ gene into TNBC cells significantly reduced Twist1 protein, but not mRNA, expression and exhibited inhibition of Twist1 activity regulating E-cadherin and N-cadherin expressions. BTG2^/TIS21^-mediated Twist1 protein loss was due to the failures of protein translation by inhibiting cap-dependent initiation via reduced mTORc1 activity and p-eIF2α maintenance, and by collapse in 80S polysome formation. That could lead to failure of protein translation at the initiation step. In addition, BTG2^/TIS21^ reduced in vivo and in vitro expressions of eukaryotic elongation factor 1 (eEF1) isoforms and eEF2. All the mechanisms could be confirmed by cDNA microarray data, RT-qPCR, and immunoblotting analyses in the TNBC cells, BTG2^/TIS21^-KO mice, and human breast cancer tissues. Taken altogether, *BTG2*^*/TIS21*^ gene inhibited the initiation and elongation steps of Twist1 translation. Thus, cancer progression including EMT phenomenon can be reduced in the cells with BTG2^/TIS21^ high expresser. In conclusion, the BTG2^/TIS21^-mediated Twist1 protein loss exhibited better prognosis in the relapse-free survival of malignant breast cancers than that in the lower BTG2^/TIS21^ and higher Twist1 expressers

We hereby presented the new role of *BTG2*^*/TIS21*^ gene as an inhibitor of Twist1 translation in the TNBC cells and the phenomenon was evidenced in tissues of the BTG2^/TIS21^-KO mice and human breast cancers (Fig. 8). To the best of our knowledge, this is the first report revealing the BTG2^/TIS21^-mediated translational downregulation of Twist1. Consequences of the BTG2^/TIS21^-mediated Twist1 loss in the TNBC cells was evidenced by the inverse regulations of N-cadherin and E-cadherin expression, which resulted in the inhibition of EMT phenomenon. EMT-inducing transcription factors, Twist1, Snail, and Zeb, are responsible for regulating oncogenesis^48^.

### Deletion of 161–202 residues of TWIST1 abrogated its interaction with BTG2

Reciprocal interaction of BTG2^/TIS21^ and C-terminal region of Twist1 was proved by in vitro IP, GST-pulldown, and deletion construct analyses (Fig. 3). The observation reminded us that Twist and p53 reciprocally regulate target genes via direct interaction^49^. Although cNOT7, a representative protein bound to BTG2 protein, and Twist1 bind to the same Box-B domain of BTG2^/TIS21^, their interaction was mutually exclusive in 293TN cells due to the differential localizations of cNOT7 and Twist1 (Supplementary Fig. S4A and S4B). Flag-cNOT7 blot for input in the Supplementary Fig. S4A shows no increase, and that may have contributed to the failure of the competitive inhibition assay in 293TN cells. However, it was competitive in HeLa cells due to their expression in the cytoplasm (Supplementary Fig. S4C–S4E). All data indicate that both Twist1 and cNOT7 bind to the same domain of BTG2^/TIS21^ protein.

### BTG2^/TIS21^ inhibited translational initiation of Twist1 mRNA

The mRNA-specific translational control can be driven by RNA sequences and/or structures in the 5′/3′-untranslated region (UTR) of the transcript^44,50^. Cap structure of mRNAs is circularized by binding to poly(A) tail via interaction of eIF4E–eIF4G–polyA-binding-protein (PABP). When the 43S preinitiation complex scans mRNAs in a 5′→3′ direction until it meets the initiation codon, the 60S ribosome subunit joins to form 80S. On the other hand, BTG2^/TIS21^ interacts with RNA-binding domain of PABPC1^51^, which can bind to N-terminal of eIF4G in the translation initiation complex^52^. The significant collapse of the polysome formation in the BTG2^/TIS21^ expresser and the loss of Twist1, but not GAPDH, mRNAs in the ribosomes (Fig. 5e, f) clearly support the inhibition of Twist1 translation by *BTG2*^*/TIS21*^ gene. Moreover, BTG2 inhibits mTORC1 activity in cancer cells^31^ and TIS21-KO MEF exhibits constitutive activation of mTORc1^53^.

In addition to failure of the cap structure formation, the BTG2^/TIS21^-mediated Twist1 loss shown in the MEF cells (Fig. 5a–d) can be regulated by maintaining eIF2α phosphorylation, the stress signal from endoplasmic reticulum^54^. When the endogenous BTG2 in MCF-7 cells was knocked down, phosphorylation of PERK was lost (evident by the band shift, please compare Lane 3 vs. Lane 1 in Supplementary Fig. S1C) and followed by eIF2α dephosphorylation. In contrast to MEF cells, BTG2^/TIS21^ expression could not alter the eIF2α phosphorylation in the TNBC cells. The discrepancy can be supported by the previous reports that phosphorylation of eIF2α is higher in human breast cancers and cells than that in the normal tissues and mammary epithelial cells^55,56^. In yeast, eIF2A functions as a suppressor of internal ribosome entry site (IRES)-mediated translation^57,58^ and regulates non-canonical translation initiation events near cognate UUG and CUG codons independent of GTP^59^. When we examined on the website RegRNA2.0^60^, there was no IRES sequences on the Twist1 mRNAs and no ribosome-binding sites on the 5′-UTR; therefore, BTG2^/TIS21^-mediated Twist1 loss might be due to the general repression of protein translation. Although further studies are required to identify more translational targets of BTG2, we here elucidated the mechanism of TWIST1 regulation by BTG2.

The BTG2^/TIS21^-mediated Twist1 loss was not due to the protein degradation by ubiquitination and autophagy reaction; in contrast, the inhibition of proteasome and lysosome rather aggravated Twist1 loss by the BTG2^/TIS21^ accumulation (Fig. 4). The data can be supported by the report that ubiquitination of BTG2^/TIS21^ by Skp2^61^. In vivo Twist1 expression was significantly higher in the TIS21-KO mice than the Wt mice (Fig. 7a, *p* < 0.01 vs. Wt), indicating the in vivo role of *BTG2*^*/TIS21*^ gene in the downregulating Twist1 translation. Considering the previous reports^23,24^ that BTG2^/TIS21^ plays in the poly(A) deadenylation by interacting with hCAF1, and that short poly(A) tails (20–50 A) repress translation as opposed to enhancement by the long tails (80–500 A)^62^, suggesting that translation regulating factors might be the potential targets of the BTG2^/TIS21^-mediated deadenylation reaction, although further evidences are required.

In eukaryotes, peptide chain elongation is mediated by the elongation factors, EF1 isoforms, and EF2^63–65^. mRNA expressions of Twist1 and the EFs were evidenced in the TNBC cells and tissues (Fig. 6, Supplementary Fig. S10). In vivo Twist1 protein expression was higher in both TIS21-KO mice and the lymph node-positive human breast cancers (Supplementary Figs. S11 and S12). Another evidence about the strong association between BTG2 expression and visceral metastatic disease in breast cancer patients (*p* < 0.001, 95% confidence interval 1.43–4.27) has been reported and it was independent of other clinico-pathologic features^66^. The findings imply the significant role of BTG2 that can prevent cancer progression via inhibition of cancer cell invasion and metastasis into surrounding tissues.

Although further analysis is needed, the present study can support our hypothesis that BTG2^/TIS21^ is a promising target to treat metastatic cancers with high TWIST1 expression by employing inhibitors to relieve epigenetic silencing of BTG2, which can prevent cancer progression from DCIS to infiltrating carcinoma.

## Materials and methods

### Cell culture

MCF-7, ZR-75-1, MDA-MB-231, MDA-MB-468, MDA-MB-453, and Hs578T cells were maintained in RPMI medium-1640 containing 10% FBS, 1% antibiotic–antimycotic solution (GIBCO, Life Technologies, Grand Island, NY) at 37 °C with 5% CO_2_ air. 293TN, HeLa, Wt MEF, and eIF2αA/A mutant MEF cells were maintained in Dulbecco’s modified Eagle medium with high glucose containing 10% FBS, 1% antibiotic–antimycotic solution (GIBCO, Life Technologies, Grand Island, NY) at 37 °C with 5% CO_2_ air. Cell lines used in our study were authenticated and tested Mycoplasma free. Short tandem repeat (STR) profiling and mycoplasma contamination of the cells used for our experiments were evaluated by AmpFlSTR PCR reaction analysis (Thermo Fisher Scientific, Waltham, MA) and e-MycoTM plus Mycoplasma PCR detection kit (iNtRON Biotech. South Korea).

### Immunoblotting

Immunoblot analysis was performed as described previously^18^. For protein expression analysis, cell lysates were separated by SDS-polyacrylamide gel electrophoresis (PAGE). Resolved proteins were transferred to nitrocellulose membrane and incubated with primary antibodies overnight at 4 °C, followed by washing and incubation with secondary antibodies. Membranes were then washed and incubated with horseradish peroxidase conjugated with anti-mouse or anti-rabbit antibodies, and the proteins were visualized by means of the enhanced chemiluminescence kit (AbClon, Inc., Seoul, Republic of Korea). Anti-Twist1, E-cadherin, N-cadherin, HA, ZEB1, BTG2 (Q-22), GAPDH, and α-tubulin antibodies were obtained from Santacruz (Dallas, TX); anti-v5 was obtained from Invitrogen (Carlsbad, CA); anti-eIF2α, Snail, eEF2, and PERK antibodies were from Cell Signaling (Danvers, MA), and anti-Flag antibody was from Sigma (St Louis, MO).

### RNA isolation and reverse transcription

For RNA isolation, samples were collected in 1.0 mL of TRIzol (Invitrogen, Carlsbad, CA) and 1.0 µg of the purified total RNAs were subjected to reverse transcription for cDNA preparation using Prime-Script reverse transcriptase (Takara, Inc., Kyoto, Japan). Extracted RNAs were stored at −80 °C and the prepared cDNAs were diluted tenfold before PCR analysis.

### Real-time PCR analysis

Real-time PCR analysis was performed to analyze the mRNA expression of various genes. Real-time quantitative RT-PCR analysis was performed in a 20 µl total volume with 2 µl diluted cDNA, 10 µl of 2× qPCR premix (RealHelixTM qPCR kit, NanoHelix, Daejeon, Korea) containing hot-start polymerase. Reaction mixtures were amplified for one cycle at 95 °C for 15 min, 50 cycles at 95 °C for 20 s and 60 °C for 40 s, and then the temperature was increased to 95 °C in 0.5 °C increments every 0.05 s along with plate reading. GAPDH and L32 were used as an internal control.

### Transduction and transfection experiments

Adenovirus carrying β-galactosidase (Ad-lacZ) or TIS21-HA (Ad-TIS21) were transduced for 48 h into MDA-MB-231 and MDA-MB-468 cells. We have used exogenous expression of TIS21 (a mouse ortholog of human BTG2) in human cancer cells. BTG2-HA and Ad-TIS21-HA plasmids contain the tag at the C-terminal end, and the constructs contain two translational start sites that make double bands in the higher percentage gels (>15%) and long separation time (~3–3.5 h). Exogenous expression was confirmed with immunoblot analysis with HA antibody. α-Tubulin served as a loading control. For siRNA transfection, siControl, or siTIS21, siBTG2 were transfected into cells using Lipofectamine 2000 reagent according to the manufacturer’s protocol. TIS21 knockdown was confirmed using real-time PCR analysis. Forced expression and knockdown experiments, transfection with siRNAs were performed 24 h before transducing the cells with adenoviruses carrying with either Ad-LacZ or Ad-TIS21. For IP analysis, 293TN cells were transfected for 24 h with plasmids either BTG2-HA, v5-Twist1, Flag-Twist1, v5-BTG2, various deletion mutants of BTG2, or Twist1. The cells were subjected to IP and immunoblot analyses.

### Serum starvation and FBS addition

To examine the regulation of Twist1 biosynthesis by the serum stimulation, MDA-MB-231 cells transduced with Ad-LacZ/Ad-TIS21 for 24 h were depleted with FBS for 18 h and then supplemented with FBS up to 1–10% for 6 h before immunoblot analysis. To further confirm the induction of Twist 1 protein biosynthesis in response to serum stimulation, the treatment time-dependent induction of Twist1 biosynthesis was tested in the MDA-MB-231 cells depleted with FBS for 18 h, and then 10% FBS was added up to 6 h before analyzed by immunoblotting.

### Subcellular fractionation

Fractionation of the 293TN and HeLa cells was performed as the adapted method from Abcam protocol (http://www.abcam.com/protocols/subcellular-fractionation-protocol).

### IP analysis

Cell lysates (1.0 mg) collected and sonicated in E1A buffer (50 mM HEPES pH 7.5, 150 mM NaCl, 0.1% NP-40, 5 mM EDTA, protease inhibitors) were incubated overnight at 4 °C with either anti-v5, anti-HA, or anti-Flag antibodies for IP analysis, and then protein G beads were added and incubated at 4 °C for 2 h before centrifugation at 1000 r.p.m. for 1 min. The beads were washed three times thoroughly and the proteins bound were eluted with 2× SDS loading buffer to examine by immunoblot analysis. Normal IgG was used as a negative control. For IP analysis, subcellular fractionation was followed and 1 mg of cytoplasmic/nuclear fractions were pulled down with 1 μg of anti-HA antibody overnight at 4 °C. Following steps were similar as mentioned above.

### Polysome profiling analysis

MDA-MB-231 and MDA-MB-468 cells transduced with Ad-LacZ/Ad-TIS21 for 48 h were treated with cycloheximide (10 µg/mL) for 10 min at room temperature, washed with cold PBS, and then lysed at cold room with 1 mL of polysome lysis buffer [20 mM HEPES pH 7.6, 5 mM MgCl_2_, 125 mM KCl, 1% NP-40, 2 mM dithiothreitol] supplemented with 100 µg/mL cycloheximide (Sigma), protease inhibitor cocktail (EDTA-free; Pierce), and RNAsin (Ambion). The cell lysates were tumbled for 15 min at 4 °C and centrifuged at 13,000 r.p.m. for 15 min. The supernatants were fractionated in 17.5–50% linear sucrose gradients by centrifugation (35,000 r.p.m. for 2 h 40 min) in a Beckman ultracentrifuge using SW40-Ti rotor. Gradients were eluted with a gradient fractionator (Brandel) and monitored with a UA-5 detector (ISCO). RNAs from each fraction were isolated and reverse transcribed as mentioned above. cDNAs were amplified using real-time PCR analysis for polysome abundance on Twist1 and GAPDH mRNAs.

### Poly(A) tail length assay

PAT assay was performed and adapted from the previous report^67^. Total RNAs were isolated from the MDA-MB-231 cells transduced with Ad-LacZ/Ad-TIS21 for 48 h. To abolish the higher ordered secondary structure at the 3′-end, RNA samples were heated at 65 °C for 5 min and immediately placed on ice. The samples were incubated for 2 h at 37 °C with 10× PAP buffer, 1 μl of PAP (1 U/μL), 1 mM MnCl_2_, and 1 mM GTP (Thermo Fisher Scientific, Waltham, MA) in a 20 µL reaction mixture. PAP buffer, PAP, and MnCl_2_ (BioVision, Milpitas, CA). Ten microliters of Guanylated RNA was used for cDNA reaction (20 μL). The first-strand cDNA synthesis was primed by oligo(dCT). As specificity control, oligo(dT) was used. The RT reaction was stopped by denaturation at 95 °C (3 min) and the cDNA product was applied to PCR amplification using Gene-specific forward and reverse primers (38 cycles) and examined by agarose gel electrophoresis.

### Isolation of mouse organs

TIS21 Wt and TIS21-KO mice under the C57BL/6 background were maintained at the Ajou University Animal Care Center under the specific pathogen-free condition with constant temperature and constant humidity. All of the animal procedures were followed by the Ajou University Institutional Review Board. Mouse organs—liver, lung, spleen, kidney, and pancreas—isolated from the TIS21-KO mice (15w–17w old) and the Wt mice (17w–20w old) were immediately snap frozen in the liquid nitrogen. Tissue samples were homogenized using a Dounce homogenizer under cold conditions (4 °C) and the samples were sonicated in RIPA buffer. Proteins (40 µg) were separated by SDS-PAGE and the resolved proteins were transferred onto nitrocellulose membrane to examine the expressions of Twist1, eEF2, and α-tubulin. For mRNA expression analysis, tissues were pulverized with liquid nitrogen and RNA extraction was performed by using TRIzol reagent. cDNAs were amplified using primers specific for eEF1α, eEF1β2, eEF1γ, eEF1ε1, and eEF2. GAPDH served as an internal control.

### Analysis of human samples

Clinical data about the breast cancer patients were collected from the Ajou University Hospital after informed consent. Tumor tissues were divided based on the degree of lymph node invasion and the matched normal tissues were obtained according to the regulations of Institutional Review Board at the Ajou University Hospital (AJIRB-GEN-SMP-11-066).

### Statistical analysis

The values presented as bar graphs are means ± SD. Statistical differences were analyzed either by Student’s *t*-test or Mann–Whitney *U*-test and the *p*-values < 0.05 were considered as statistically significant.

## Supplementary information

Supplementary Figure Legends

Supplementary Figures
