# Supplementary material for: Translational downregulation of Twist1 expression by antiproliferative gene, B-cell translocation gene 2, in the triple negative breast cancer cells
Source: Cell Death Dis. 2019 May 28;10(6):410. doi: 10.1038/s41419-019-1640-z (PMC6538657; doi:10.1038/s41419-019-1640-z)
Supplement: Supplementary file 1 — Supplementary Figure Legends [file 41419_2019_1640_MOESM1_ESM.docx]

**SUPPLEMENTARY FIGURE LEGENDS:**

**Supplementary Figure S1. Endogenous expressions of BTG2^/TIS21^ in various breast cancer cells:**

(**A**) Total RNAs were isolated from the cells (3 × 10^5^ cells/60mm) incubated for 24 h, and then subjected to RT-PCR using BTG2-specific primers. GAPDH was used as a control. Note the loss of BTG2 expression in the highly invasive breast cancer cells MDA-MB-231, MDA-MB-468, MDA-MB-453, and HS578T as compared with other cell lines. (**B**) To evaluate whether the exogenously expressed BTG2^/TIS21^ is physiologically relevant to the endogenous BTG2 or not, wt-MEF, MCF-7, ZR-75-1 and TNBC cells (transduced with either Ad-LacZ or Ad-BTG2^/TIS21^ for 48h) were subjected to immunoblot analysis and examined the expression of BTG2 and α- Tubulin. Blots are representative of two independent experiments. Note similar level of BTG2^/TIS21^ expression in the TNBC cells transduced with 100 moi of Ad-TIS21 as compared with the endogenous BTG2^/TIS21^ level in the MEF, MCF-7 and ZR-75-1 cells. (**C**) MCF-7 cells were transfected with siControl and siBTG2 in a dose dependant manner (25nM, 50nM) for 48h, and total RNA and protein were isolated before subjected to RT-PCR and immunoblot analysis. RT-PCR was performed by amplifying Twist1 and BTG2 genes. GAPDH was used as a control. Immunoblot was performed with antibodies against TWIST1, PERK, p-eIF2α, eIF2α, eEF2 and α-tubulin. Blots are representative of two independent experiments.

**Supplementary Figure S2. Forced expression of BTG2^/TIS21^ downregulated Twist1 protein expression in the TNBC cells:**

(**A**) To investigate effect of BTG2^/TIS21^ gene on the Twist1 expression, adenovirus carrying LacZ or BTG2^/TIS21^ gene was transduced to MDA-MB-231 and MDA-MB-468 cells in a dose dependent manner. In 48 h, the cell lysates were subjected to immunoblot analysis. Note downregulation of Twist1 protein expression in a dose dependent manner of BTG2^/TIS21^ gene. Transduction of Ad-BTG2^/TIS21^ was evaluated by anti-HA antibody. α-Tubulin served as a loading control. Blots are representative of three independent experiments. (**B**) To evaluate whether the BTG2^/TIS21^ effect on Twist1 expression is specific or not, TNBC cells transduced with either Ad-LacZ or Ad-BTG2^/TIS21^ for 48h were subjected to RT-qPCR analysis and examined expression of IL6 mRNAs. Note significant inhibition of IL6 mRNA expression in the BTG2^/TIS21^ expresser than that in the LacZ control. GAPDH expression served as internal control of transcription. Data are expressed as mean ± SD for two independent experiment. **(C, D)** To validate inhibition of Twist1 protein, but not mRNAs, expression by BTG2^/TIS21^ gene in TNBC cells, MDA-MB-231 cells were transduced with the adenoviruses for 24 h and then cultured without serum for 18h before re-stimulation with 10% FBS for 6 h. Note the serum concentration-dependent (**C**) and the treatment time-dependent (D) induction of Twist1 proteins in the LacZ control as opposed to strong suppression in the BTG2^/TIS21^ expresser. α-Tubulin was used as a loading control. Blots are representative of two independent experiment.

**Supplementary Figure S3.** **Poly(A) tail length (PAT) Assay:**

MDA-MB-231 cells (3 × 10^5^ cells/60mm) were transduced with Ad-LacZ/Ad-TIS21 (100moi) for 48h, and total RNAs were isolated before subjected to PAT assay. RT-PCR was performed using Twist1-tail specific and gene specific primers. GAPDH was used as a control. OligodT served as a specificity control (please note lanes 3 and 4 in the upper panel) for our analysis. Blots are representative of two independent experiments.

**Supplementary Figure S4. C-terminal region of Twist1 interacts with BTG2^/TIS21^ protein.**

(**A**) BTG2^/TIS21^ binding to Twist1 was not interfered by the BTG2-cNOT7 interaction in 293TN cells. To evaluate whether the BTG2^/TIS21^-cNOT7 interaction can disturb the BTG2^/TIS21^ binding to Twist1, competitive inhibition assay was performed in 293TN cells overexpressed with BTG2-HA, v5-Twist1 and Flag-cNOT7. The cell lysates were subjected to immunoprecipitation with anti-HA (1.0 µg) antibody and then immunoblot analyses with anti-HA, anti-v5, anti-cNOT7, and anti-Flag antibodies. Interaction of BTG2-HA with v5-Twist1 was not interfered by the overexpression of cNOT7, since most of Twist1 protein was expressed in nuclei of 293TN cells. IgG served as a negative control. (**B**) Differential localization of cNOT7 and Twist1 expression in 293TN cells. The cells overexpressed with BTG2-HA and v5-Twist1 were fractionated into whole cell lysates (WCL), cytoplasm, and nucleus, and then subjected to immunoblot analysis (40 µg/lane). Endogenous cNOT7 protein was expressed only in the cytoplasm, whereas v5-Twist1 was mainly found in the nuclei. α-Tubulin and histone-H3 were used as the marker of cytoplasm and nucleus, respectively. (**C**) Intracellular localization of Twist1, cNOT7 and BTG2 in HeLa cells. The cells overexpressed with BTG2-HA, v5-Twist1 and Flag-CNOT7 were fractionated into whole cell lysates (WCL), cytoplasm and nucleus, and each fraction (40µg) was subjected to immunoblot analysis. Flag-cNOT7 was expressed only in the cytoplasm, and v5-Twist1 was mainly expressed in the cytoplasm and less amount in nucleus. BTG2 was found in both cytoplasm and nuclei of HeLa cells. GAPDH and histone-H3 were used as the marker of cytoplasm and nucleus, respectively. (**D**) Confocal imaging analysis revealing the co-localization of v5-Twist1 and BTG2-HA in the cytoplasm of HeLa cells. (**E**) Competition assay was performed in HeLa cells overexpressed with BTG2-HA, v5-Twist1 and Flag-cNOT7. The cell lysates were subjected to immunoprecipitation with anti-HA (1.0 µg) antibody and then immunoblot analyses with anti-HA, anti-v5 and anti-Flag antibodies. Interaction of BTG2-HA with v5-Twist1 was interfered by the expression of cNOT7, since most of Twist1 was expressed in the cytoplasm of HeLa cells. IgG served as a negative control. Blots are representative of two independent experiment. (**F-H**) Prediction of the intrinsically unstructured protein was searched by using (*https://iupred2a.elte.hu/*) software, and the disorder tendency of the Twist1, BTG2 and TIS21 proteins was represented. Note the structured domain of Twist1 only in the C-terminal (121-201) residues as opposed to the BTG2 and TIS21 proteins structured in the whole regions.

**Supplementary Figure S5. Endogenous BTG2 interacts with TWIST1 and with cNOT7 in MCF-7 cells:**

To examine the endogenous BTG2 activity in interaction with TWIST1 and cNOT7 proteins, MCF-7 cells were employed. (**A**) Cells were transfected with v5-Twist1, and then immunoprecipitation was performed with anti-BTG2 (1.0 µg) antibody, and followed by immunoblot analysis with anti-BTG2, anti-v5, and anti-cNOT7 antibodies. Note endogenous BTG2 protein interacted with these proteins. (**B**) Intracellular localization of TWIST1, cNOT7 and BTG2 proteins in MCF-7 cells were examined. The cells transfected with v5-Twist1 were fractionated into whole cell lysates (WCL), cytoplasm, and nucleus. The each fraction (40µg) was subjected to immunoblot analysis. Endogenous cNOT7 was expressed only in the cytoplasm, v5-Twist1 and BTG2 was found in both cytoplasm and nuclear fractions. α-Tubulin and histone-H3 were used as the marker of cytoplasm and nucleus, respectively. Blots are representative of two independent experiment.

**Supplementary Figure S6. BTG2^/TIS21^-mediated Twist1 loss is independent of ubiquitination of Twist1 protein.**

(**A**) To explore whether BTG2^/TIS21^**-**mediated Twist1 loss is due to the proteasome activation or not, transfection of MDA-MB-231 cells with vector, 3xFlag-Twist1, Ub-HA with or without v5-BTG2^/TIS21^ were performed and then the cells were analysed by IP with anti-Flag antibody and then immunoblot with anti-Flag, anti-v5 and anti-HA antibodies to detect the change of ubiquitination. Note absence of the increased ubiquitination of Twist1 protein in the BTG2-HA expresser despite their interaction. (**B**) As a positive control of the ubiquitination study, ABI-2 protein expression was employed. MDA-MB-231 cells transfected with vector, v5-ABI-2, Ub-HA with or without Flag-TIS21 were immunoprecipitated with anti-v5 antibody and then immunoblotted with anti-Flag, anti-v5 and anti-HA antibodies to detect the change of ubiquitination. Note presence of partial increase in the ubiquitination of ABI-2 protein in the BTG2-HA expresser along with their interaction. Blots are representative of two independent experiment.

**Supplementary Figure S7. Phosphorylation of 4EBP1 and level of eEF2 expression are decreased in the TNBC cells with BTG2^/TIS21^ overexpression.** To examine whether there is any interaction between BTG2^/TIS21^ and eIF2α proteins, MDA-MB-231 (**A**) and MDA-MB-468 (**B**) cells were analysed by immunoprecipitation with anti-HA antibody after transduction with either Ad-LacZ or Ad-BTG2^/TIS21^ for 48h, and then followed by immunoblot with anti-eIF2α and anti-HA antibodies. Note absence of the interaction between BTG2^/TIS21^ and eIF2α in both cells. IgG was used as a negative control. (**C**) Downregulation of 4EBP1 phosphorylation at the S^65^ and T^37/46^ residues in MDA-MB-231 cells with BTG2^/TIS21^ expression. The total 4EBP1 level was not changed and α-tubulin was used as a loading control. (**D**) No change in the phosphorylation of 4EBP1 at T^70^ residue and p-eIF2α in the TNBC cells with BTG2^/TIS21^ expression, however, expression of eEF2 was completely lost in the TNBC cells with BTG2^/TIS21^ expression. α-Tubulin was used as a loading control. Blots are representative of two independent experiments.

**Supplementary Figure S8. Microarray data reveal the increased expression of translational initiation factors in the TIS21-KO mice compared to those in the wild type**

Heatmap presentation of the eukaryotic translation factors regulating initiation, elongation and termination steps retrieved from our data (NCBI GEO as the GSE105772). Note the statistically upregulated genes in the TIS21-KO mice compared to those in the WT mice.

**Supplementary Figure S9. Treatment of MDA-MB-231 cells with rapalogs significantly inhibited TWIST1 expression in MDA-MB-231 cells.**

**(A**) Twist1 mRNAs in human and mice were examined, and we searched presence of 5’TOP motifs conserved in the two species. (**B**) MDA-MB-231 cells were treated with Rapamycin, Torin 1 and PP242 for 6 and 12h, and then subjected to immunoblot analysis. The treatment of MDA-MB-231 cells with rapalogs significantly inhibited TWIST1 expression in 12 h. p-p70S6K levels were measured to monitor activity of the drugs. Blots are representative of two independent experiment.

**Supplementary Figure S10. Expression of eEFs is also significantly higher in the BTG2^/TIS21^-KO mice** **than the wild type:**

Animal body (**A**) and organ (**B**) weights in the BTG2^/TIS21^-KO and the wild type female mice. There was no effect of TIS21-knockout on the weights. mRNA expressions of the eukaryotic elongation factors in liver (**C**), lung (**D**), and spleen (**E**) of the mice were determined by real time PCR with GAPDH as an internal control. Expressions of eEFs were significantly higher in the BTG2^/TIS21^-KO mice than those in wild type. p<0.05 was considered as statistically significant. (**F**) There was no expression of BTG2^/TIS21^ gene in the organs of the BTG2^/TIS21^-KO mice. (**G**) Twist1 mRNA expression measured by real time PCR was not significantly changed between the BTG2^/TIS21^-KO and wild type mice. GAPDH served as an internal control for amplification.

**Supplementary Figure S11. *In vivo* expression of Twist1 protein is higher in the BTG2^/TIS21^-KO mice than the wild type**

To confirm *in vivo* effect of BTG2^/TIS21^-KO on Twist1 expression, several organs were extracted from the TIS21-Wt and TIS21-KO female mice (n=6 mice in each group) and then analysed by immunoblotting (40µg/lane). Protein expression of Twist1 was significantly increased in the lung and spleen of TIS21-KO mice than that in the wild type. In liver, pancreas and kidney, Twist1 expression was not significantly different between the KO and wild type mice. α-Tubulin represents protein loading control and each lane shows animal variation.

**Supplementary Figure S12. Expression of Twist1, SNAIL and eEF2 is significantly higher in human breast cancers with lymph node metastasis, whereas BTG2 expression is observed only in the normal tissues.**

(**A**) Human breast cancer tissues with and without lymph node invasion (stage 0 and stage 2, respectively) were analysed by immunoblot to examine protein expressions of Twist1, Snail, eEF2 and GAPDH. The expressions were much stronger in the node stage 2 tumours compared to those in the stage 0. N is the surrounding normal tissue, T indicates tumour tissue. (**B**) RT-PCR analysis showing BTG2 mRNA levels in the lymph node positive breast tumours and surrounding normal tissues. Note absence of BTG2 expression in tumour (T) as opposed to the expression in normal (N) tissues. L-32 is used as an internal control.
