# Supplementary material for: Translational downregulation of Twist1 expression by antiproliferative gene, B-cell translocation gene 2, in the triple negative breast cancer cells
Source: Cell Death Dis. 2019 May 28;10(6):410. doi: 10.1038/s41419-019-1640-z (PMC6538657; doi:10.1038/s41419-019-1640-z)
Supplement: Supplementary file 2 — Supplementary Figures [file 41419_2019_1640_MOESM2_ESM.pptx]

## Slide 1
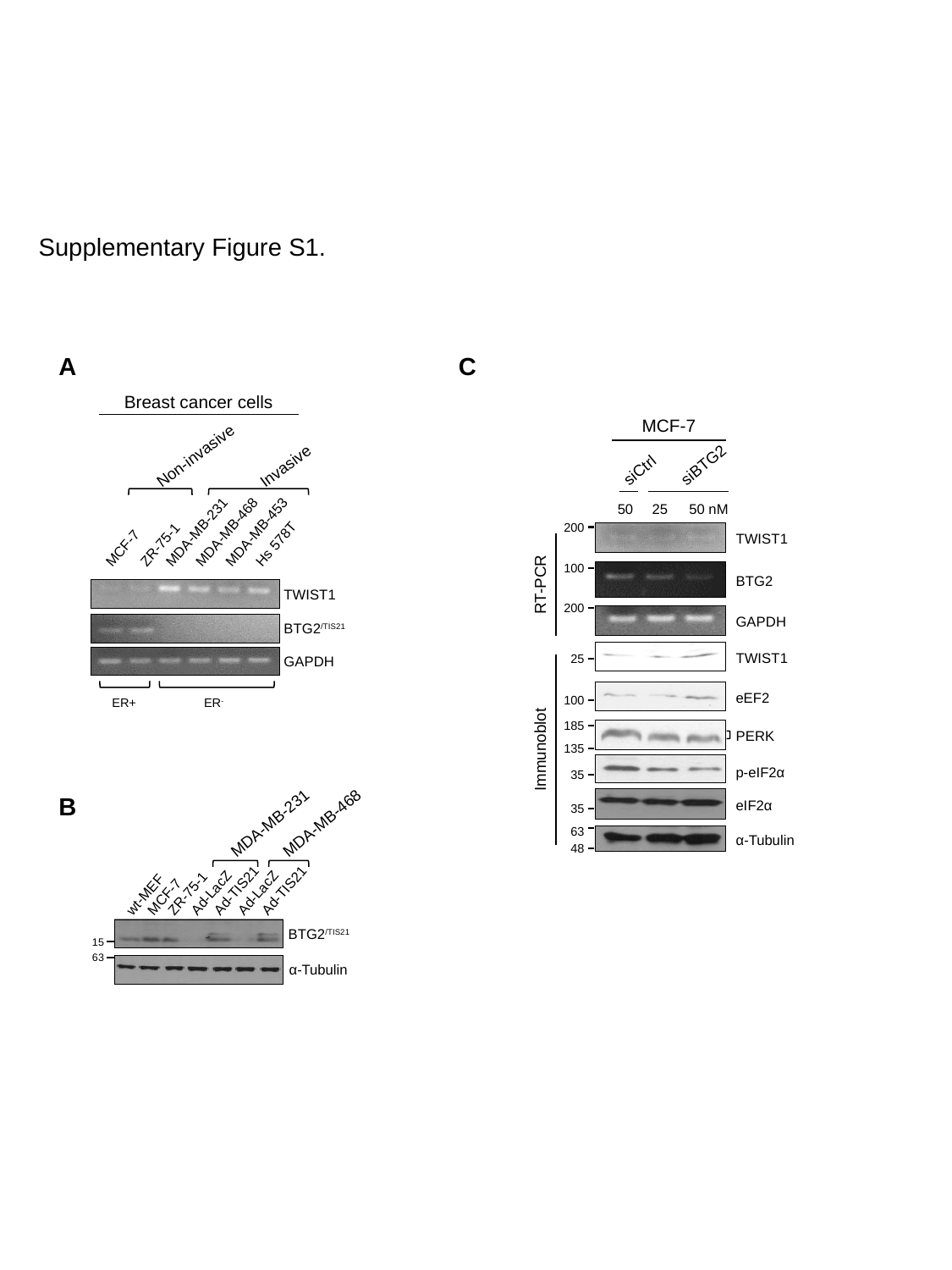

Supplementary Figure S1.
A
C
Breast cancer cells
Non-invasive
Invasive
MDA-MB-468
MDA-MB-453
MDA-MB-231
Hs 578T
ZR-75-1
MCF-7
TWIST1
BTG2/TIS21
GAPDH
ER+
ER-
MCF-7
siBTG2
siCtrl
50
25
50 nM
200
TWIST1
100
BTG2
RT-PCR
200
GAPDH
TWIST1
25
eEF2
100
185
PERK
Immunoblot
135
p-eIF2α
35
eIF2α
35
63
α-Tubulin
48
B
MDA-MB-231
MDA-MB-468
wt-MEF
Ad-LacZ
Ad-TIS21
Ad-LacZ
Ad-TIS21
ZR-75-1
MCF-7
BTG2/TIS21
15
63
α-Tubulin

## Slide 2
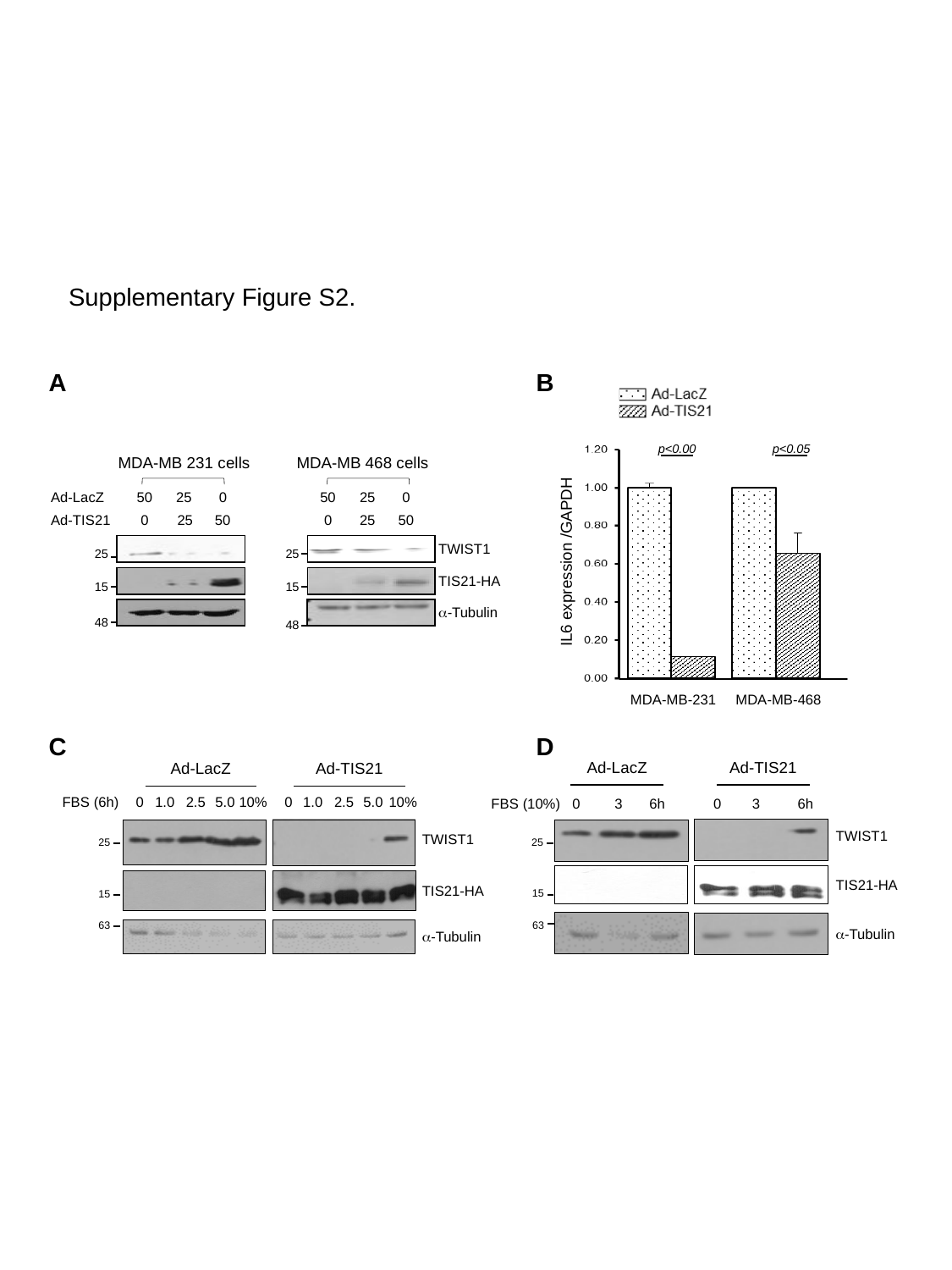

Supplementary Figure S2.
A
B
p<0.00
p<0.05
 IL6 expression /GAPDH
MDA-MB-231
MDA-MB-468
MDA-MB 231 cells
MDA-MB 468 cells
Ad-LacZ
50
25
0
50
25
0
Ad-TIS21
0
25
50
0
25
50
TWIST1
25
25
TIS21-HA
15
15
α-Tubulin
48
48
C
D
Ad-LacZ
Ad-TIS21
FBS (10%)
0
3
6h
0
3
6h
Twist1
25
TIS21-HA
15
63
α-Tubulin
Ad-LacZ
Ad-TIS21
FBS (6h)
0
1.0
2.5
5.0
10%
0
1.0
2.5
5.0
10%
Twist1
25
TIS21-HA
15
63
α-Tubulin

## Slide 3
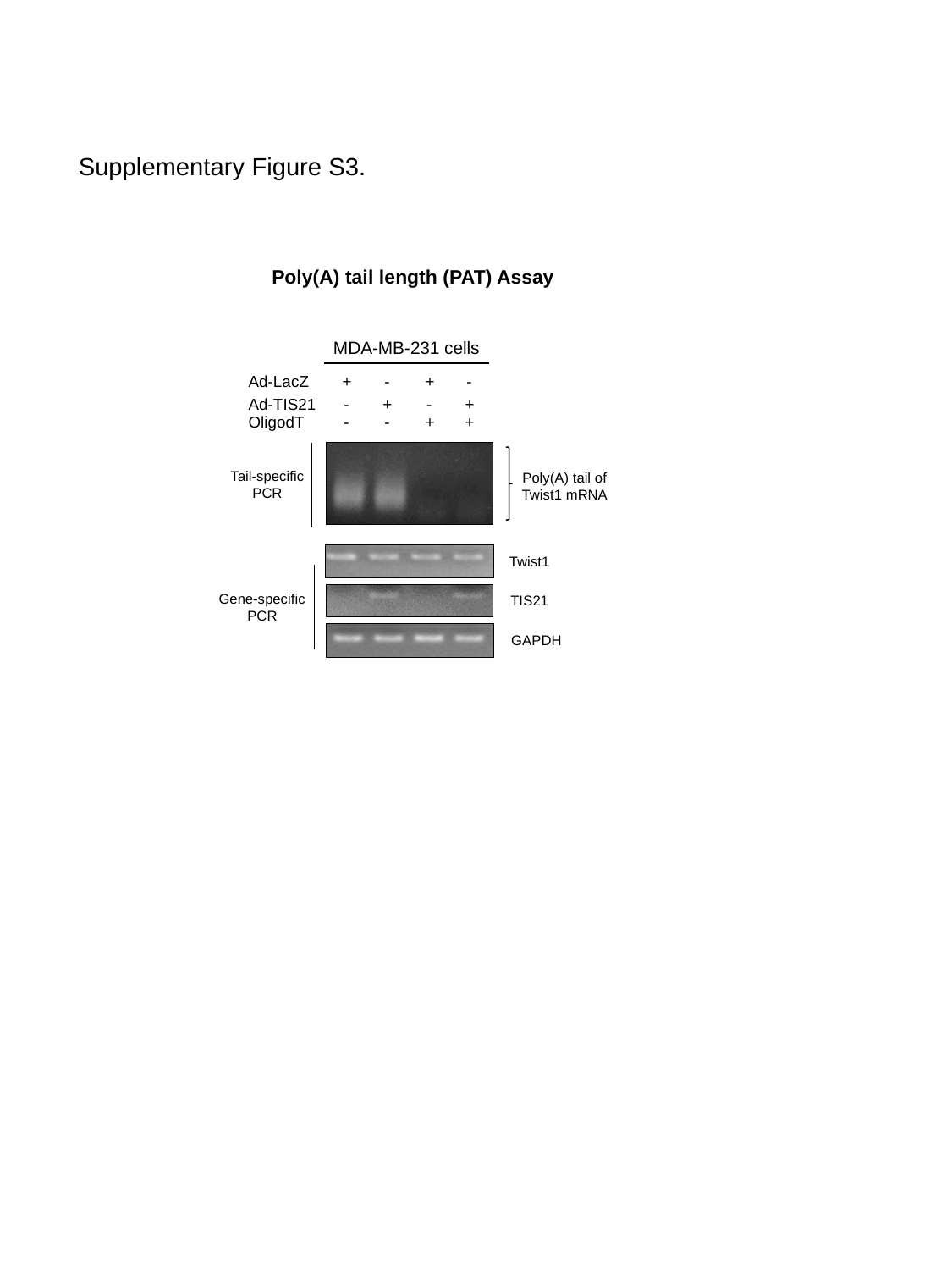

Supplementary Figure S3.
Poly(A) tail length (PAT) Assay
MDA-MB-231 cells
Ad-LacZ
+
+
-
-
Ad-TIS21
-
-
+
+
OligodT
-
+
+
-
Tail-specific PCR
Poly(A) tail of Twist1 mRNA
Twist1
Gene-specific PCR
TIS21
GAPDH

## Slide 4
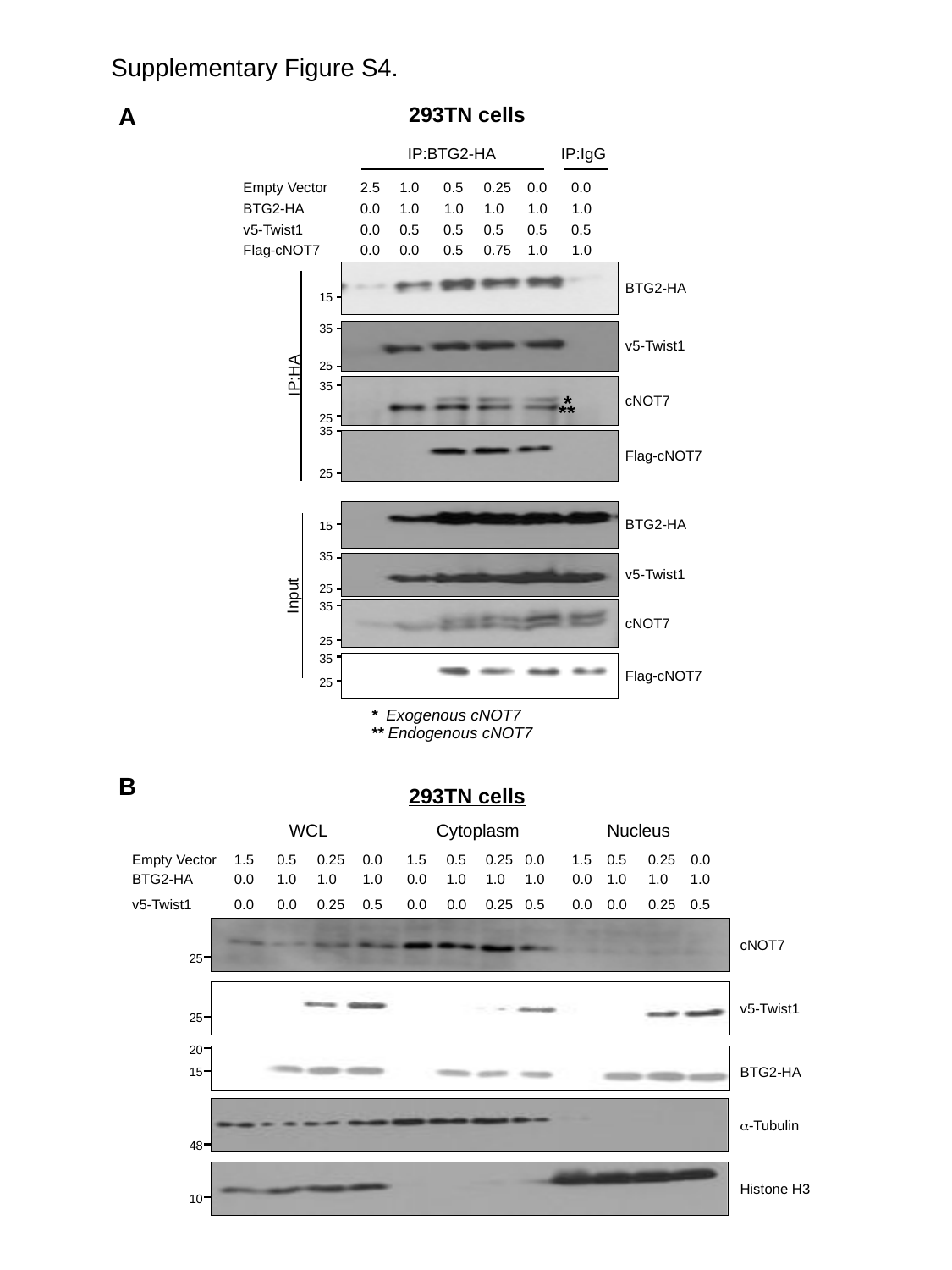

Supplementary Figure S4.
A
293TN cells
IP:BTG2-HA
IP:IgG
Empty Vector
2.5
1.0
0.5
0.25
0.0
0.0
BTG2-HA
0.0
1.0
1.0
1.0
1.0
1.0
v5-Twist1
0.0
0.5
0.5
0.5
0.5
0.5
Flag-cNOT7
0.0
0.0
0.5
0.75
1.0
1.0
BTG2-HA
15
35
v5-Twist1
25
IP:HA
35
*
cNOT7
**
25
35
Flag-cNOT7
25
BTG2-HA
15
35
v5-Twist1
25
Input
35
cNOT7
25
35
Flag-cNOT7
25
* Exogenous cNOT7
** Endogenous cNOT7
B
293TN cells
WCL
Cytoplasm
Nucleus
1.5
0.5
0.25
0.0
1.5
0.5
0.25
0.0
1.5
0.5
0.25
0.0
Empty Vector
0.0
1.0
1.0
1.0
0.0
1.0
1.0
1.0
0.0
1.0
1.0
1.0
BTG2-HA
0.0
0.0
0.25
0.5
0.0
0.0
0.25
0.5
0.0
0.0
0.25
0.5
v5-Twist1
cNOT7
25
v5-Twist1
25
20
BTG2-HA
15
α-Tubulin
48
Histone H3
10

## Slide 5
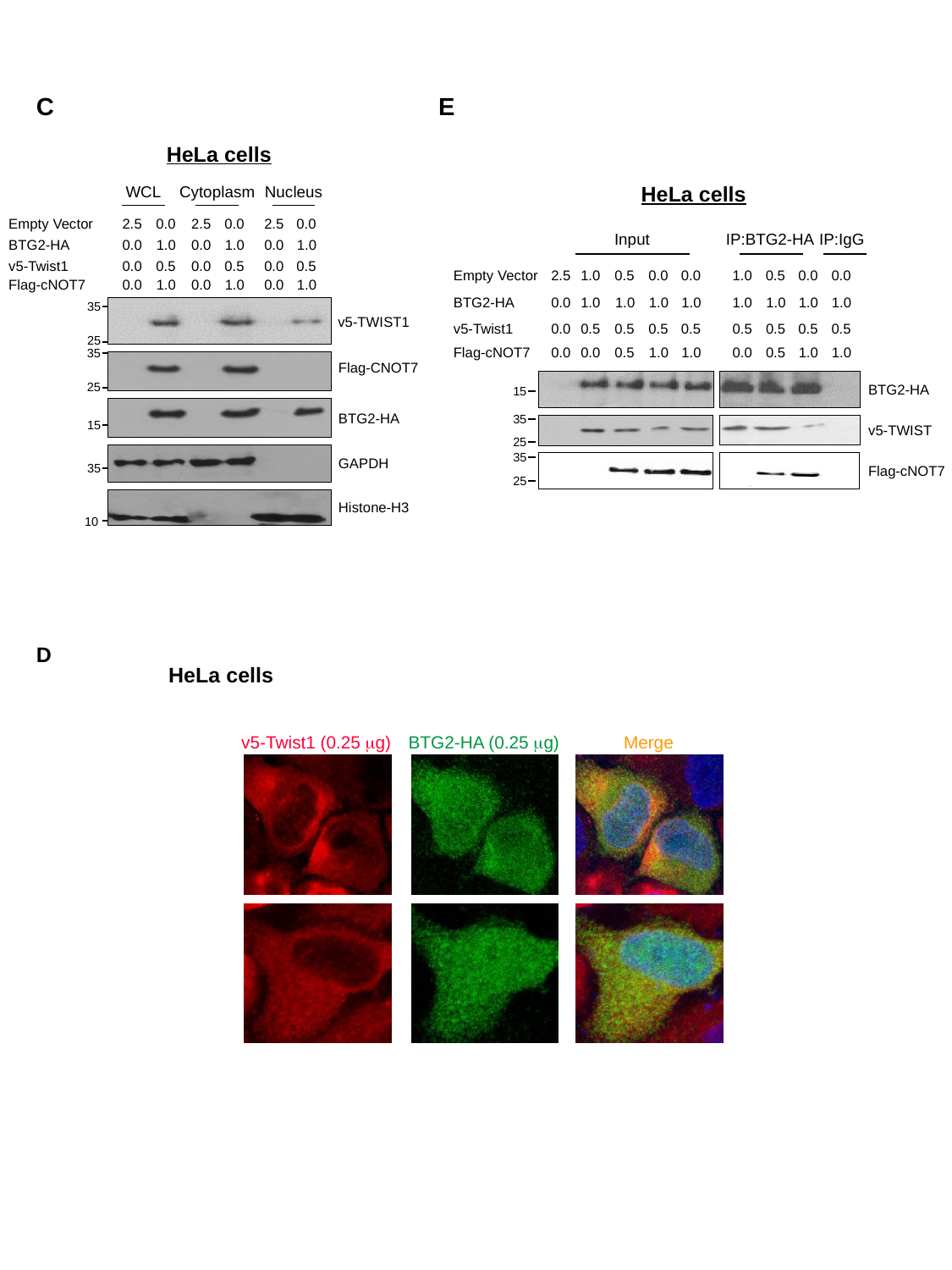

C
E
HeLa cells
WCL
Cytoplasm
Nucleus
Empty Vector
2.5
0.0
2.5
0.0
2.5
0.0
BTG2-HA
0.0
1.0
0.0
1.0
0.0
1.0
v5-Twist1
0.0
0.5
0.0
0.5
0.0
0.5
Flag-cNOT7
0.0
1.0
0.0
1.0
0.0
1.0
35
v5-TWIST1
25
35
Flag-CNOT7
25
BTG2-HA
15
GAPDH
35
Histone-H3
10
HeLa cells
Input
IP:BTG2-HA
IP:IgG
Empty Vector
2.5
1.0
0.5
0.0
0.0
1.0
0.5
0.0
0.0
BTG2-HA
0.0
1.0
1.0
1.0
1.0
1.0
1.0
1.0
1.0
v5-Twist1
0.0
0.5
0.5
0.5
0.5
0.5
0.5
0.5
0.5
Flag-cNOT7
0.0
0.0
0.5
1.0
1.0
0.0
0.5
1.0
1.0
BTG2-HA
15
35
v5-TWIST
25
35
Flag-cNOT7
25
D
HeLa cells
v5-Twist1 (0.25 mg)
BTG2-HA (0.25 mg)
Merge

## Slide 6
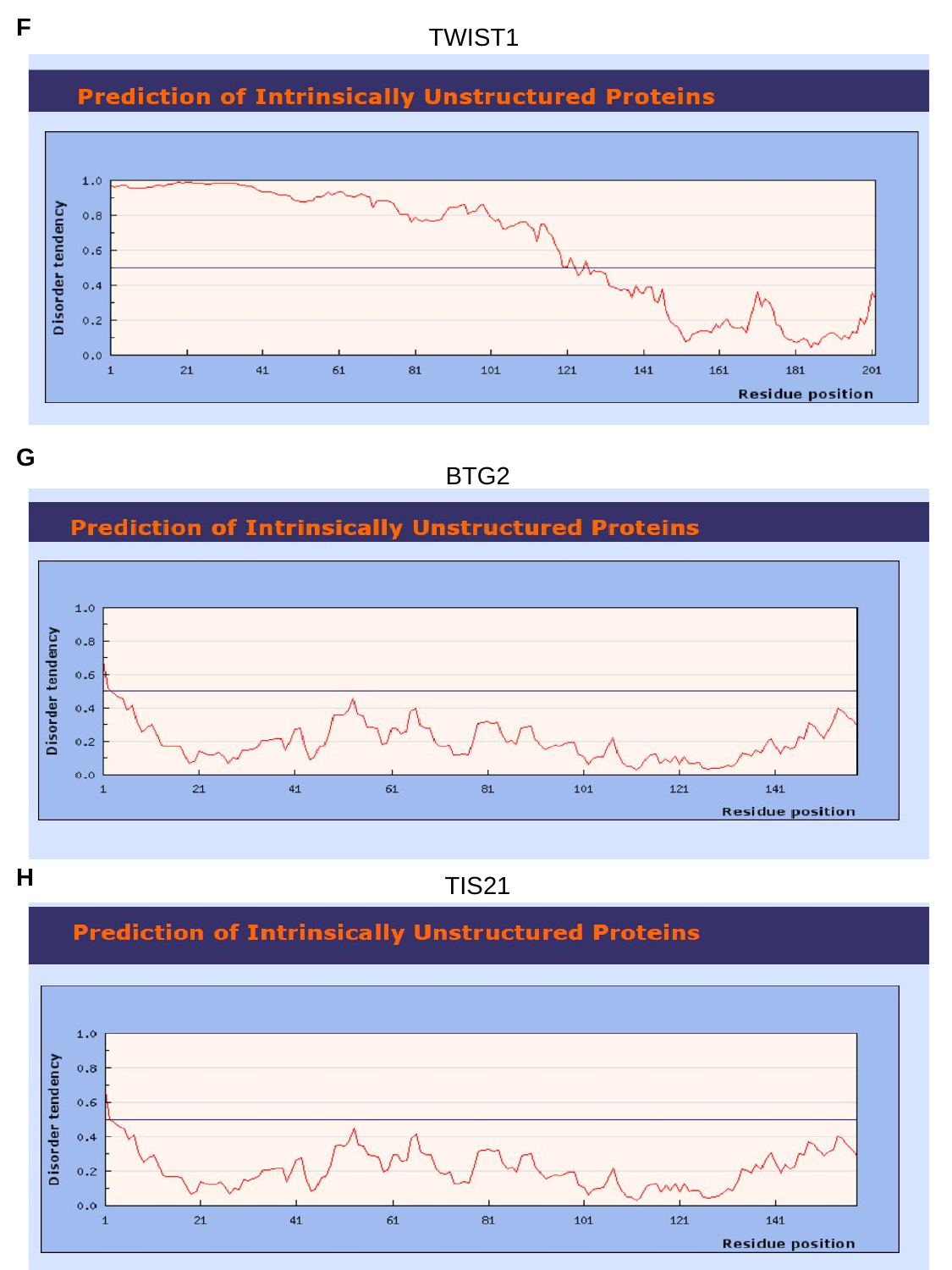

F
TWIST1
G
BTG2
H
TIS21

## Slide 7
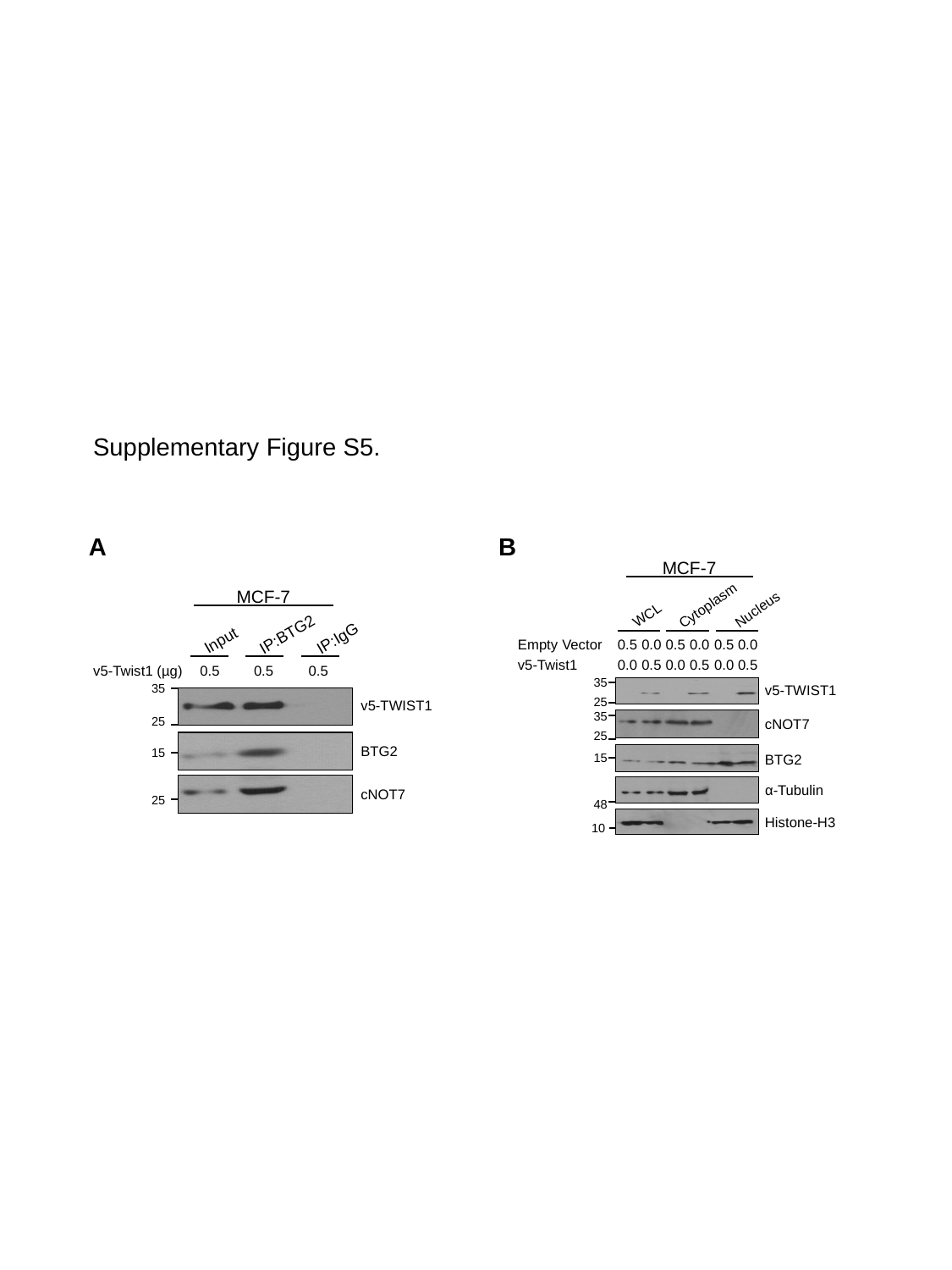

Supplementary Figure S5.
A
B
MCF-7
Cytoplasm
Nucleus
WCL
Empty Vector
0.5
0.0
0.5
0.0
0.5
0.0
v5-Twist1
0.0
0.5
0.0
0.5
0.0
0.5
35
v5-TWIST1
25
35
cNOT7
25
15
BTG2
α-Tubulin
48
Histone-H3
10
MCF-7
IP:BTG2
IP:IgG
Input
v5-Twist1 (µg)
0.5
0.5
0.5
35
v5-TWIST1
25
BTG2
15
cNOT7
25

## Slide 8
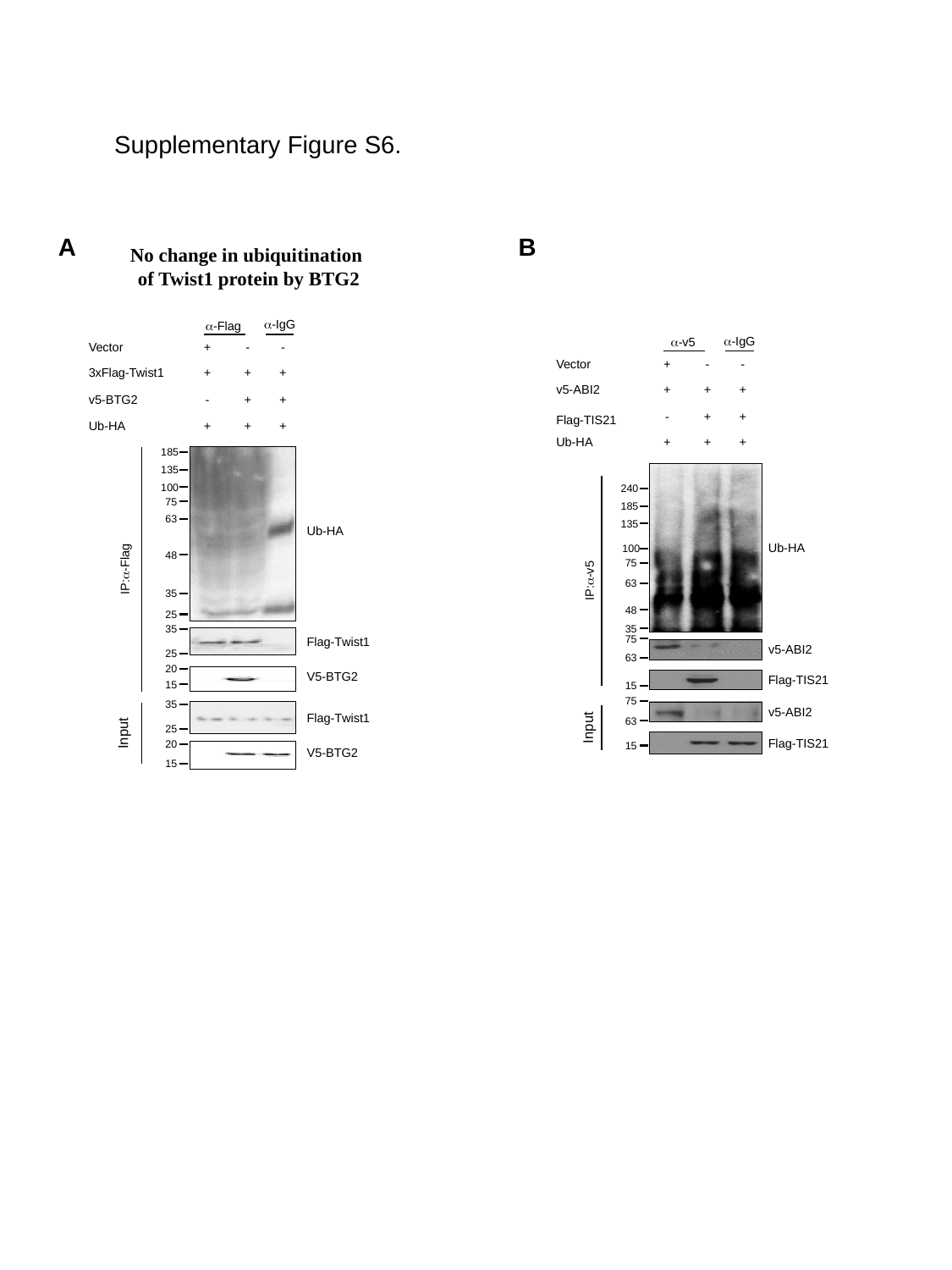

Supplementary Figure S6.
A
B
No change in ubiquitination
of Twist1 protein by BTG2
a-IgG
a-Flag
Vector
+
-
-
3xFlag-Twist1
+
+
+
v5-BTG2
-
+
+
Ub-HA
+
+
+
185
135
100
75
63
Ub-HA
48
IP:a-Flag
35
25
35
Flag-twist1
25
20
V5-BTG2
15
35
Flag-twist1
Input
25
20
V5-BTG2
15
a-IgG
a-v5
Vector
+
-
-
v5-ABI2
+
+
+
-
+
+
Flag-TIS21
Ub-HA
+
+
+
240
185
135
Ub-HA
100
75
IP:a-v5
63
48
35
75
v5-ABI2
63
Flag-TIS21
15
75
v5-ABI2
63
Input
Flag-TIS21
15

## Slide 9
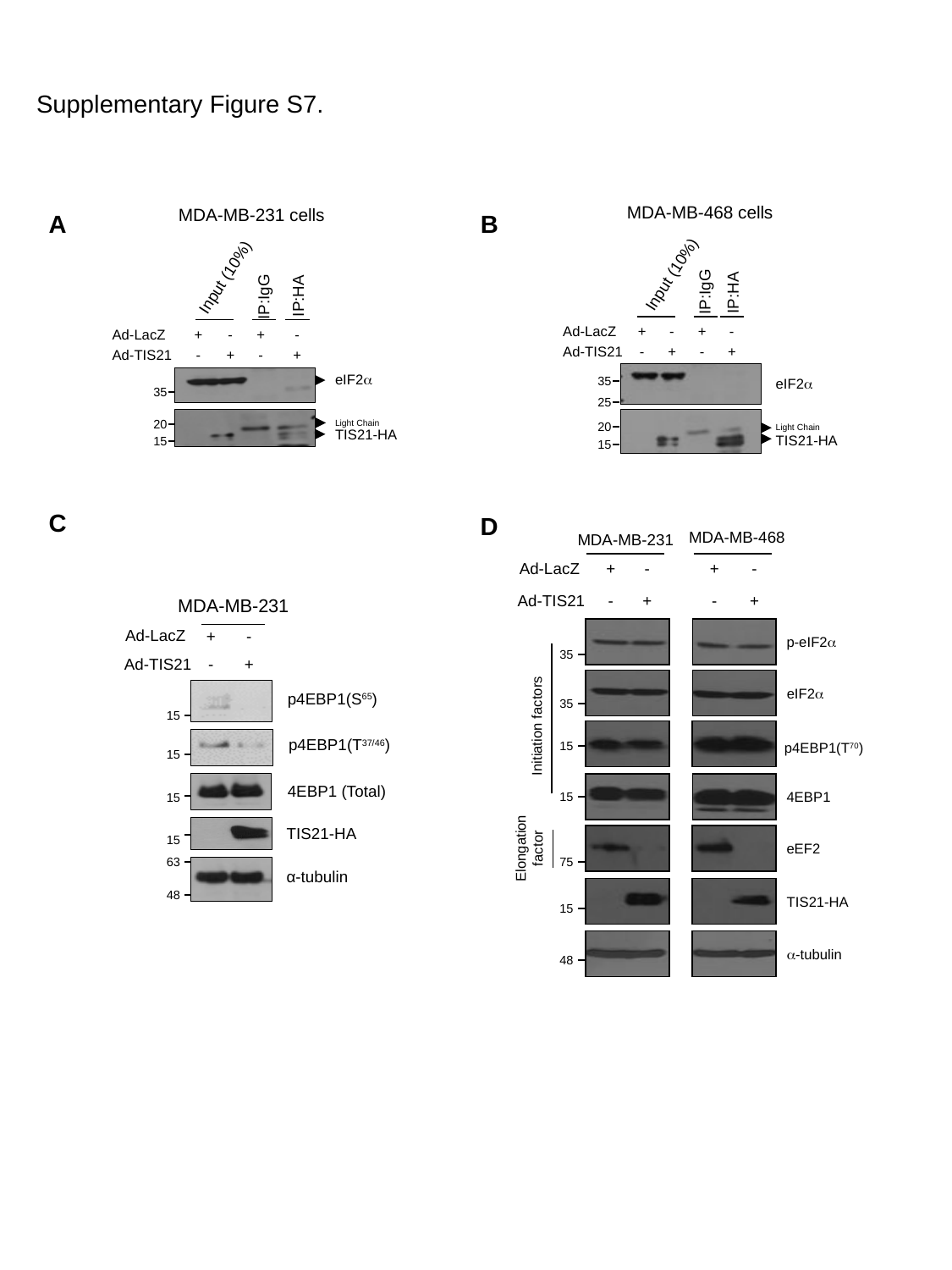

Supplementary Figure S7.
MDA-MB-468 cells
MDA-MB-231 cells
A
B
Input (10%)
IP:IgG
IP:HA
Ad-LacZ
+
-
+
-
Ad-TIS21
-
+
-
+
35
eIF2α
25
20
Light Chain
TIS21-HA
15
Input (10%)
IP:HA
IP:IgG
Ad-LacZ
+
-
+
-
Ad-TIS21
-
+
-
+
eIF2α
35
20
Light Chain
TIS21-HA
15
C
D
MDA-MB-468
MDA-MB-231
Ad-LacZ
+
-
+
-
Ad-TIS21
-
+
-
+
p-eIF2α
35
eIF2α
35
Initiation factors
15
p4EBP1(T70)
4EBP1
15
Elongation
factor
eEF2
75
TIS21-HA
15
α-tubulin
48
MDA-MB-231
Ad-LacZ
+
-
Ad-TIS21
-
+
p4EBP1(S65)
15
p4EBP1(T37/46)
15
4EBP1 (Total)
15
TIS21-HA
15
63
α-tubulin
48

## Slide 10
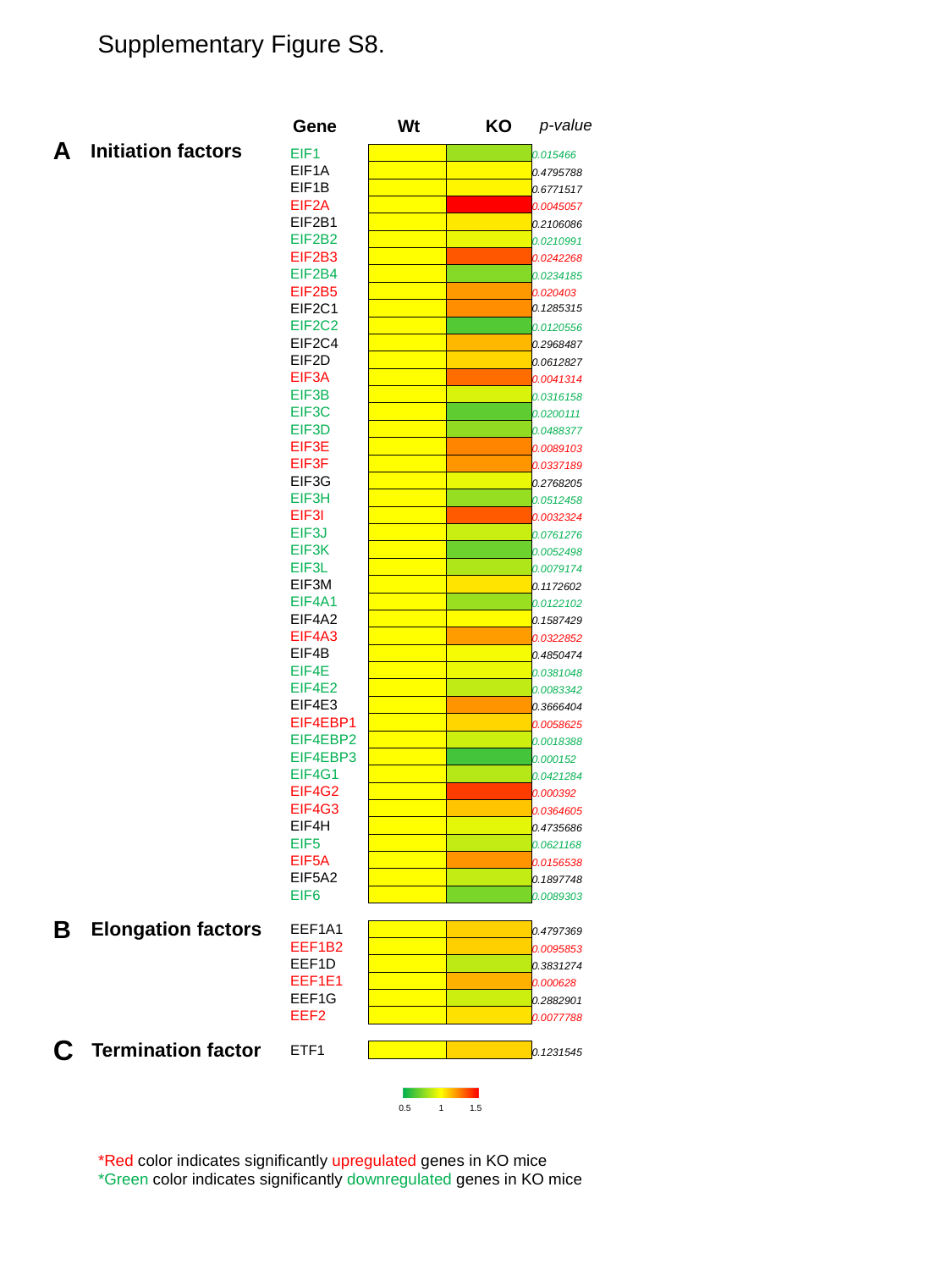

Supplementary Figure S8.
Gene
p-value
Wt
KO
A
Initiation factors
| EIF1 | | | 0.015466 |
| --- | --- | --- | --- |
| EIF1A | | | 0.4795788 |
| EIF1B | | | 0.6771517 |
| EIF2A | | | 0.0045057 |
| EIF2B1 | | | 0.2106086 |
| EIF2B2 | | | 0.0210991 |
| EIF2B3 | | | 0.0242268 |
| EIF2B4 | | | 0.0234185 |
| EIF2B5 | | | 0.020403 |
| EIF2C1 | | | 0.1285315 |
| EIF2C2 | | | 0.0120556 |
| EIF2C4 | | | 0.2968487 |
| EIF2D | | | 0.0612827 |
| EIF3A | | | 0.0041314 |
| EIF3B | | | 0.0316158 |
| EIF3C | | | 0.0200111 |
| EIF3D | | | 0.0488377 |
| EIF3E | | | 0.0089103 |
| EIF3F | | | 0.0337189 |
| EIF3G | | | 0.2768205 |
| EIF3H | | | 0.0512458 |
| EIF3I | | | 0.0032324 |
| EIF3J | | | 0.0761276 |
| EIF3K | | | 0.0052498 |
| EIF3L | | | 0.0079174 |
| EIF3M | | | 0.1172602 |
| EIF4A1 | | | 0.0122102 |
| EIF4A2 | | | 0.1587429 |
| EIF4A3 | | | 0.0322852 |
| EIF4B | | | 0.4850474 |
| EIF4E | | | 0.0381048 |
| EIF4E2 | | | 0.0083342 |
| EIF4E3 | | | 0.3666404 |
| EIF4EBP1 | | | 0.0058625 |
| EIF4EBP2 | | | 0.0018388 |
| EIF4EBP3 | | | 0.000152 |
| EIF4G1 | | | 0.0421284 |
| EIF4G2 | | | 0.000392 |
| EIF4G3 | | | 0.0364605 |
| EIF4H | | | 0.4735686 |
| EIF5 | | | 0.0621168 |
| EIF5A | | | 0.0156538 |
| EIF5A2 | | | 0.1897748 |
| EIF6 | | | 0.0089303 |
| | | | |
| EEF1A1 | | | 0.4797369 |
| EEF1B2 | | | 0.0095853 |
| EEF1D | | | 0.3831274 |
| EEF1E1 | | | 0.000628 |
| EEF1G | | | 0.2882901 |
| EEF2 | | | 0.0077788 |
| | | | |
| ETF1 | | | 0.1231545 |
B
Elongation factors
C
Termination factor
0.5
1
1.5
*Red color indicates significantly upregulated genes in KO mice
*Green color indicates significantly downregulated genes in KO mice

## Slide 11
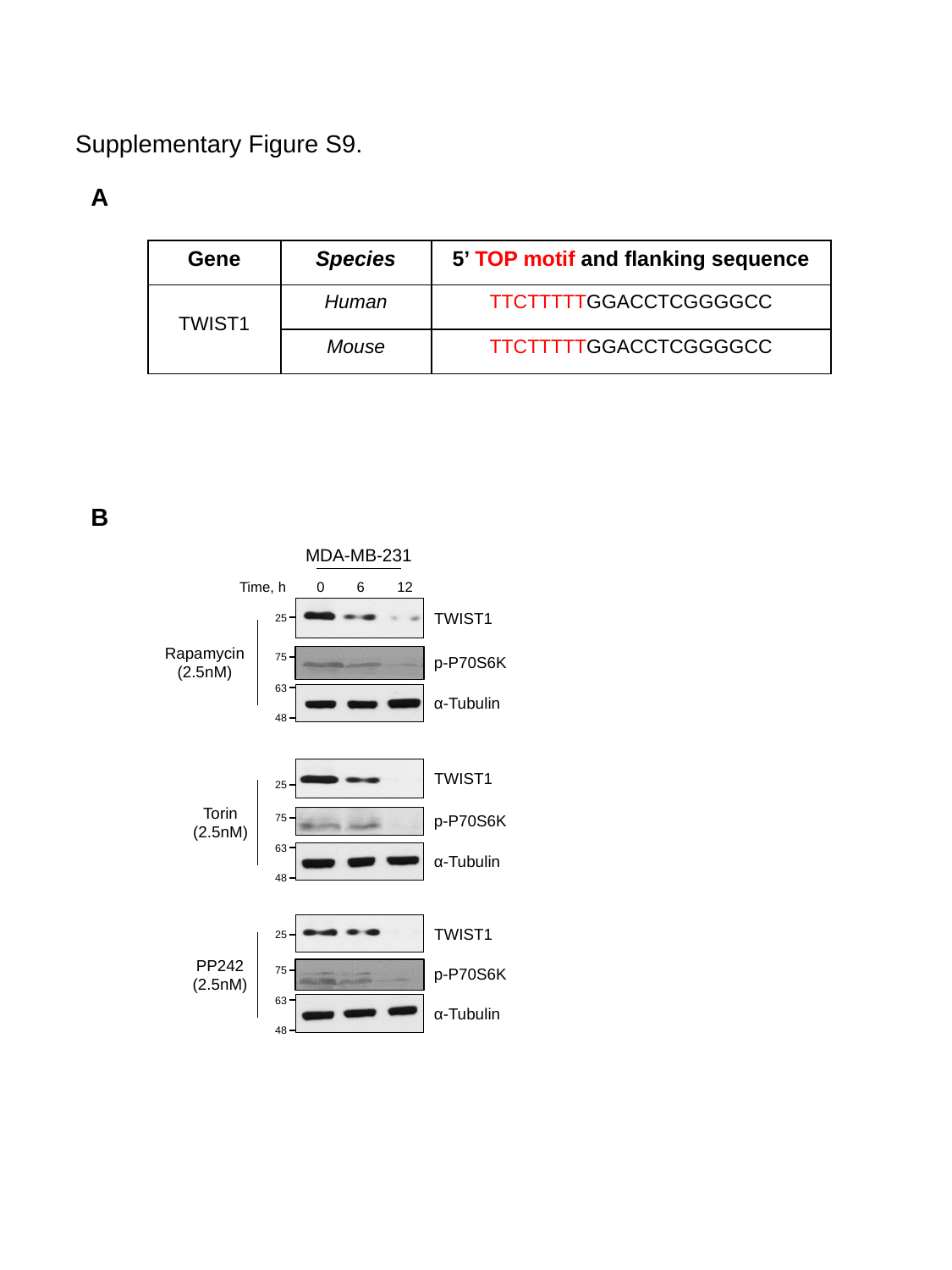

Supplementary Figure S9.
A
| Gene | Species | 5’ TOP motif and flanking sequence |
| --- | --- | --- |
| TWIST1 | Human | TTCTTTTTGGACCTCGGGGCC |
| | Mouse | TTCTTTTTGGACCTCGGGGCC |
B
MDA-MB-231
Time, h
0
6
12
TWIST1
25
Rapamycin (2.5nM)
75
p-P70S6K
63
α-Tubulin
48
TWIST1
25
Torin (2.5nM)
75
p-P70S6K
63
α-Tubulin
48
TWIST1
25
PP242 (2.5nM)
75
p-P70S6K
63
α-Tubulin
48

## Slide 12
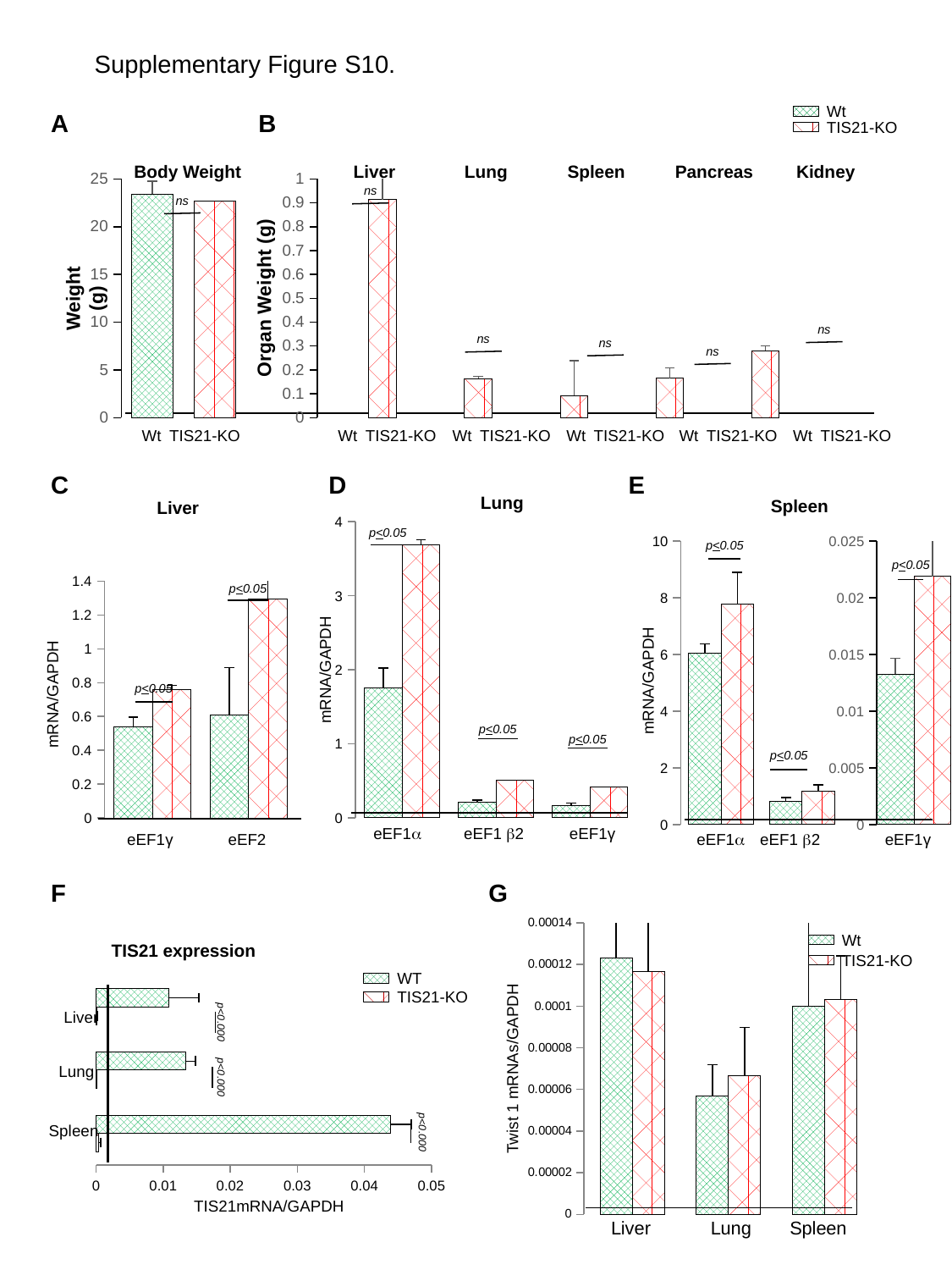

Supplementary Figure S10.
Wt
TIS21-KO
Pancreas
Body Weight
Liver
Lung
Spleen
Kidney
### Chart
| Category | |
|---|---|
### Chart
| Category | | |
|---|---|---|ns
ns
Weight (g)
Organ Weight (g)
ns
ns
ns
ns
Wt
TIS21-KO
Wt
TIS21-KO
Wt
TIS21-KO
Wt
TIS21-KO
Wt
TIS21-KO
Wt
TIS21-KO
A
B
C
D
E
Lung
### Chart
| Category | | |
|---|---|---|p<0.05
mRNA/GAPDH
p<0.05
p<0.05
eEF1α
eEF1 β2
eEF1γ
Spleen
### Chart
| Category | | |
|---|---|---|
### Chart
| Category | |
|---|---|p<0.05
p<0.05
mRNA/GAPDH
p<0.05
eEF1α
eEF1γ
eEF1 β2
Liver
### Chart
| Category | | |
|---|---|---|p<0.05
p<0.05
mRNA/GAPDH
eEF1γ
eEF2
F
G
### Chart
| Category | | |
|---|---|---|Wt
TIS21-KO
Twist 1 mRNAs/GAPDH
Liver
Lung
Spleen
TIS21 expression
WT
### Chart
| Category | | |
|---|---|---|
| Spleen | 0.0003133333333333333 | 0.04388333333333333 |
| Lung | 7.000000000000001e-05 | 0.013406666666666666 |
| Liver | 8.666666666666668e-05 | 0.010823333333333332 |
TIS21-KO
Liver
p<0.000
Lung
p<0.000
Spleen
p<0.000
TIS21mRNA/GAPDH

## Slide 13
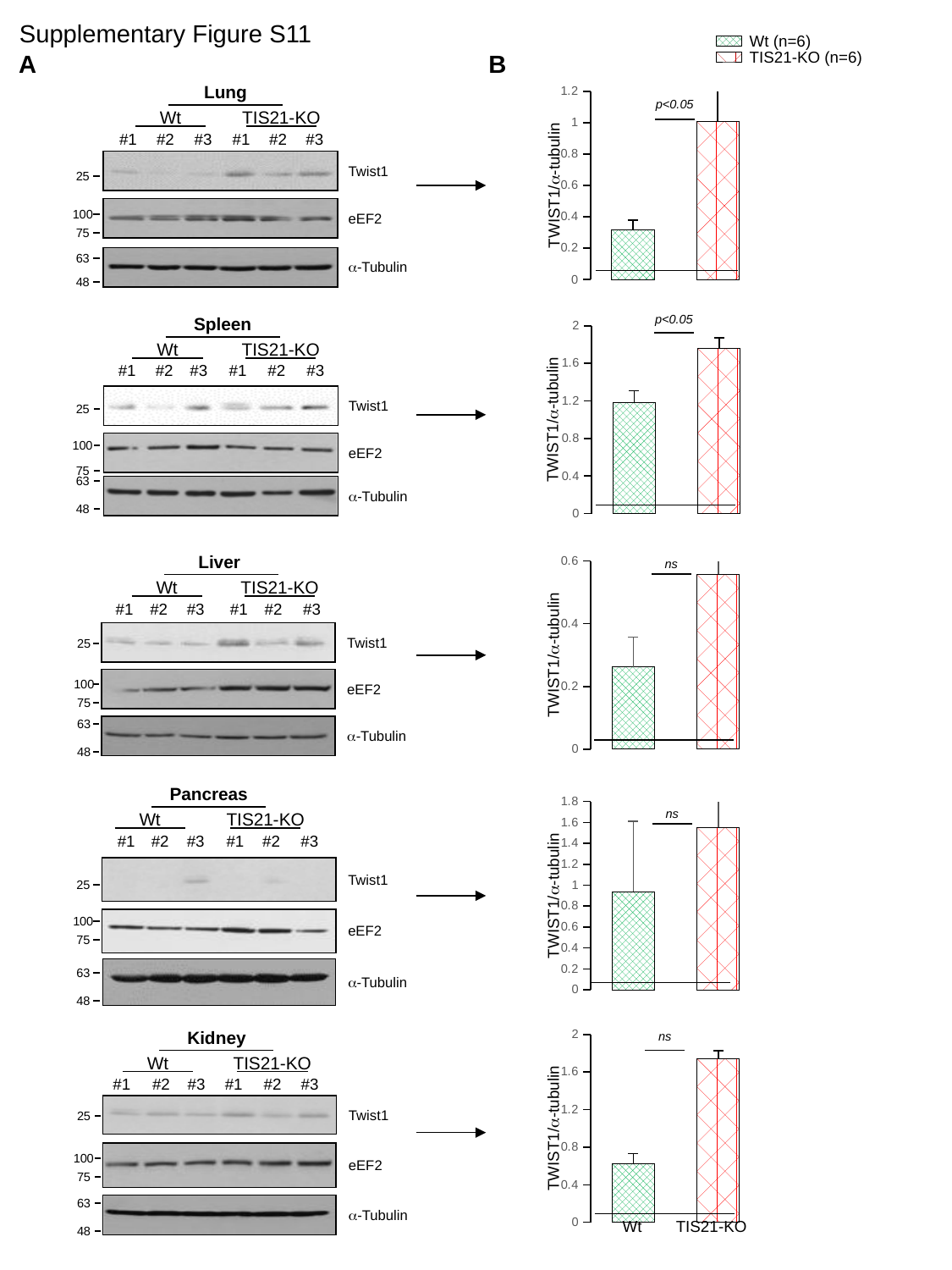

Supplementary Figure S11
Wt (n=6)
TIS21-KO (n=6)
A
B
Lung
Wt
TIS21-KO
#1
#2
#3
#1
#2
#3
Twist1
25
100
eEF2
75
63
α-Tubulin
48
### Chart
| Category | |
|---|---|p<0.05
Twist1/α-tubulin
p<0.05
### Chart
| Category | |
|---|---|Twist1/α-tubulin
Spleen
Wt
TIS21-KO
#1
#2
#3
#1
#2
#3
Twist1
25
100
eEF2
75
63
α-Tubulin
48
Liver
Wt
TIS21-KO
#1
#2
#3
#1
#2
#3
Twist1
25
100
eEF2
75
63
α-Tubulin
48
ns
### Chart
| Category | |
|---|---|Twist1/α-tubulin
Pancreas
Wt
TIS21-KO
#1
#2
#3
#1
#2
#3
Twist1
25
100
eEF2
75
63
α-Tubulin
48
### Chart
| Category | |
|---|---|ns
Twist1/α-tubulin
Kidney
Wt
TIS21-KO
#1
#2
#3
#1
#2
#3
Twist1
25
100
eEF2
75
63
α-Tubulin
48
ns
### Chart
| Category | |
|---|---|Twist1/α-tubulin
Wt
TIS21-KO

## Slide 14
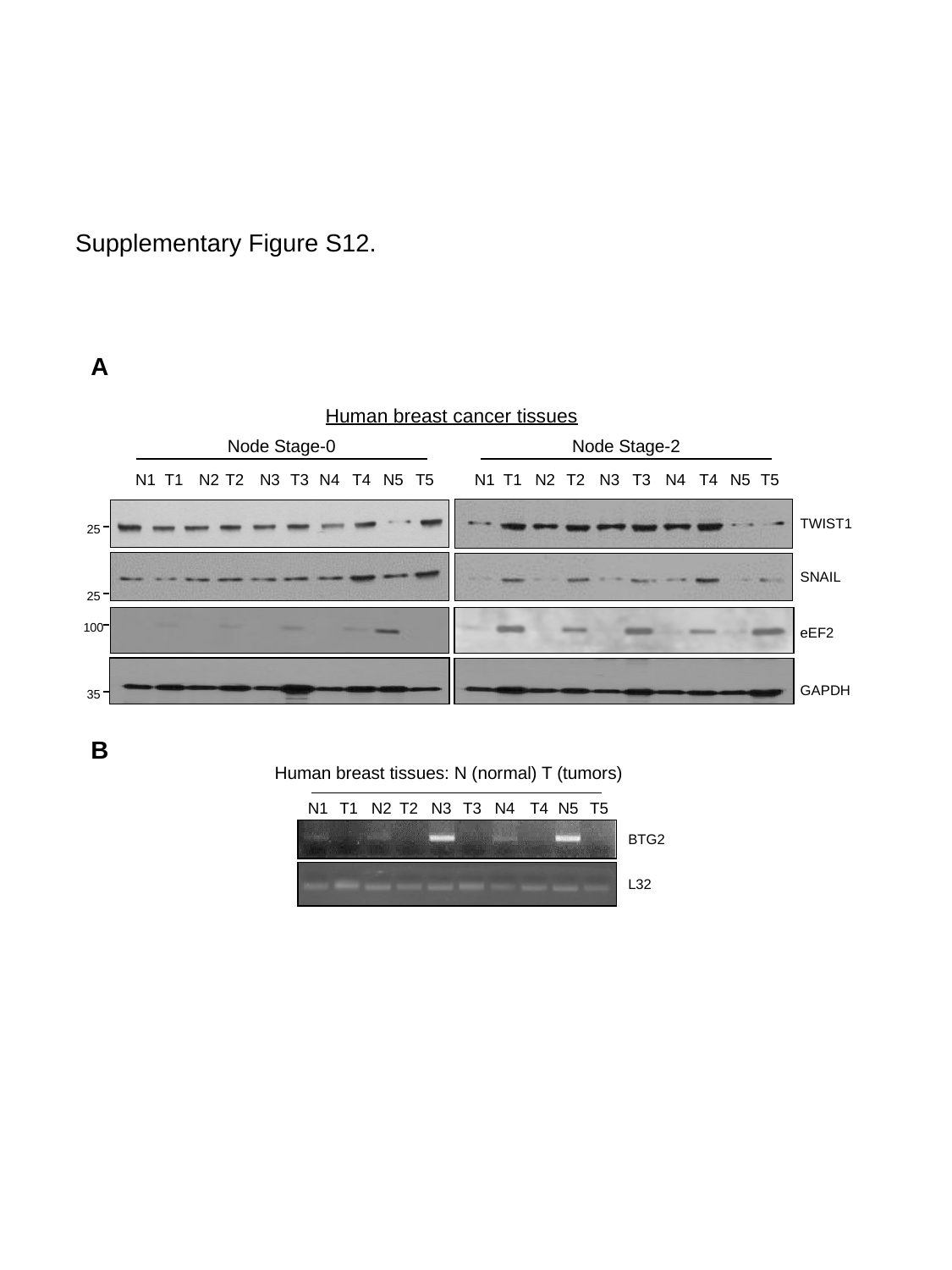

Supplementary Figure S12.
A
Human breast cancer tissues
Node Stage-0
Node Stage-2
N1
T1
N2
T2
N3
T3
N4
T4
N5
T5
N1
T1
N2
T2
N3
T3
N4
T4
N5
T5
Twist1
25
Snail
25
100
eEF2
GAPDH
35
B
Human breast tissues: N (normal) T (tumors)
N1
T1
N2
T2
N3
T3
N4
T4
N5
T5
BTG2
L32
